# Burden of diarrhea and antibiotic use among children in low-resource settings preventable by *Shigella* vaccination: A simulation study

**DOI:** 10.1371/journal.pmed.1004271

**Published:** 2023-11-22

**Authors:** Stephanie A. Brennhofer, James A. Platts-Mills, Joseph A. Lewnard, Jie Liu, Eric R. Houpt, Elizabeth T. Rogawski McQuade

**Affiliations:** 1 Division of Infectious Diseases & International Health, University of Virginia, Charlottesville, Virginia, United States of America; 2 Division of Epidemiology, School of Public Health, University of California, Berkeley, California, United States of America; 3 School of Public Health, Qingdao University, Qingdao, Shandong, People’s Republic of China; 4 Department of Epidemiology, Rollins School of Public Health, Emory University, Atlanta, Georgia, United States of America

## Abstract

**Background:**

*Shigella* is a leading cause of diarrhea and dysentery in children in low-resource settings, which is frequently treated with antibiotics. The primary goal of a *Shigella* vaccine would be to reduce mortality and morbidity associated with *Shigella* diarrhea. However, ancillary benefits could include reducing antibiotic use and antibiotic exposures for bystander pathogens carried at the time of treatment, specifically for fluoroquinolones and macrolides (F/M), which are the recommended drug classes to treat dysentery. The aim of the study was to quantify the reduction in *Shigella* attributable diarrhea, all diarrhea, and antibiotic use in the first 2 years of life that could be prevented by a *Shigella* vaccine.

**Methods and findings:**

We used data from the Etiology, Risk Factors, and Interactions of Enteric Infections and Malnutrition and the Consequences for Child Health and Development (MAL-ED) study, a birth cohort study that followed 1,715 children with twice weekly surveillance for enteric infections, illnesses, and antibiotic use for the first 2 years of life from November 2009 to February 2014 at 8 sites. We estimated the impact of 2 one-dose (6 or 9 months) and 3 two-dose (6 and 9 months, 9 and 12 months, and 12 and 15 months) *Shigella* vaccines on diarrheal episodes, overall antibiotic use, and F/M use. Further, we considered additional protection through indirect and boosting effects. We used Monte Carlo simulations to estimate the absolute and relative reductions in the incidence of diarrhea and antibiotic use comparing each vaccination scenario to no vaccination. We analyzed 9,392 diarrhea episodes and 15,697 antibiotic courses among 1,715 children in the MAL-ED birth cohort study. There were 273.8 diarrhea episodes, 30.6 shigellosis episodes, and 457.6 antibiotic courses per 100 child-years. A *Shigella* vaccine with a mean vaccine efficacy of 60% against severe disease given at 9 and 12 months prevented 10.6 (95% CI [9.5, 11.5]) *Shigella* diarrhea episodes of any severity per 100 child-years (relative 34.5% reduction), 3.0 (95% CI [2.5, 3.5]) F/M courses for *Shigella* treatment per 100 child-years (relative 35.8% reduction), and 5.6 (95% CI [5.0, 6.3]) antibiotic courses of any drug class for *Shigella* treatment per 100 child-years (relative 34.5% reduction). This translated to a relative 3.8% reduction in all diarrhea, a relative 2.8% reduction in all F/M courses, a relative 3.1% reduction in F/M exposures to bystander pathogens, and a relative 0.9% reduction in all antibiotic courses. These results reflect *Shigella* incidence and antibiotic use patterns at the 8 MAL-ED sites and may not be generalizable to all low-resource settings.

**Conclusions:**

Our simulation results suggest that a *Shigella* vaccine meeting WHO targets for efficacy could prevent about a third of *Shigella* diarrhea episodes, antibiotic use to treat shigellosis, and bystander exposures due to shigellosis treatment. However, the reductions in overall diarrhea episodes and antibiotic use are expected to be modest (<5%).

## Abstract 2

Leveraging data from 1715 children in the MAL-ED birth cohort study, Stephanie A Brennhofer and colleagues model the expected reduction in diarrhea episodes, antibiotic use, and bystander pathogen exposure to antibiotics through the administration of Shigella vaccines.

## Author summary

**Why was this study done?:**

- *Shigella* is a leading cause of diarrhea and dysentery among children under 5 years of age in low- and middle- income countries (LMICS).
- There are several *Shigella* vaccines in the pipeline poised to reduce mortality and moderate-to-severe diarrhea episodes.
- The World Health Organization (WHO) has specified that a preferred *Shigella* vaccine would have a minimum of 60% vaccine efficacy against moderate-to-severe diarrhea, be 1 to 2 doses, and the series would be completed before 12 months of age.
- WHO has encouraged estimating antibiotic use as a secondary endpoint in *Shigella* vaccine trials.

**What did the researchers do and find?:**

- We performed Monte Carlo simulations using data from 1,715 children in the Etiology, Risk Factors, and Interactions of Enteric Infections and Malnutrition and the Consequences for Child Health and Development (MAL-ED) birth cohort study to estimate the expected reduction in diarrhea episodes, antibiotic use, and bystander pathogen exposure to antibiotics through the administration of 1- and 2-dose *Shigella* vaccines given at varying ages to coincide with vaccines currently administrated in the Expanded Programme on Immunization (EPI) schedule.
- Vaccine efficacy was simulated using a beta distribution with a mean vaccine efficacy of 60% or 80% for severe diarrhea.
- A 2-dose *Shigella* vaccine with 60% efficacy given at 9 and 12 months could reduce 34.5% of *Shigella* diarrhea episodes, 34.5% of antibiotic courses to treat *Shigella*, and 33.9% of antibiotic exposures to bystander pathogens to treat shigellosis.
- A 2-dose *Shigella* vaccine with 60% efficacy given at 9 and 12 months could reduce 3.8% of diarrhea episodes of any etiology, 0.9% of antibiotic courses for diarrhea overall, and 1.1% of all antibiotic exposures to bystander pathogens.

**What do these findings mean?:**

- A *Shigella* vaccine could considerably reduce *Shigella*-associated diarrhea and antibiotic use but will likely have a modest effect on all-cause diarrhea and antibiotic use.
- The MAL-ED study sites may not reflect the expected impact of a *Shigella* vaccine in all low-resource settings.
- Future studies should account for varying vaccine efficacy by *Shigella* species and serotypes and consider waning immunity over a longer follow-up period.

## Introduction

*Shigella* is a leading cause of diarrhea and dysentery in children under the age of 5 in low- and middle-income countries (LMICs) [1]. In the multisite Etiology, Risk Factors, and Interactions of Enteric Infections and Malnutrition and the Consequences for Child Health and Development (MAL-ED) study, a prospective birth cohort conducted in Dhaka, Bangladesh; Fortaleza, Brazil; Vellore, India; Bhaktapur, Nepal; Naushero Feroze, Pakistan; Loreto, Peru; Venda, South Africa; and Haydom, Tanzania, the incidence of *Shigella*-attributed diarrhea was 26.1 episodes per 100 child-years in the first 2 years of life [2]. *Shigella* is the second leading cause of diarrhea mortality [3] after rotavirus and has been associated with intestinal inflammation [4] and short-term decrements in height [5,6]. A high burden of *Shigella* infections and *Shigella*-attributable diarrhea has been further associated with decrements in linear growth at age 2 [5], with effects persisting to 5 years [5] and 6 to 8 years [7].

Furthermore, previous analyses have identified *Shigella* as a leading contributor to antibiotic consumption for diarrhea among children in low-resource settings [8], which has implications for antibiotic resistance. In MAL-ED, *Shigella* was responsible for 14.8 antibiotic courses per 100 child-years, which accounts for 11.7% of all antibiotic courses for diarrhea in the first 2 years of life [9]. Of all fluoroquinolone and macrolide courses given for diarrhea, 20.9% (attributable incidence: 3.09 per 100 child-years; 95% CI [2.64, 3.66]) and 16.2% (attributable incidence: 4.59; 95% CI [3.92, 5.47]), respectively, were to treat shigellosis [9]. Frequent use of antibiotics drives selection for drug-resistant pathogens [10], and drug-resistant shigellosis is of concern [11]. Azithromycin- and fluoroquinolone-resistant strains of *Shigella* are common in Asia and are growing in prevalence elsewhere [12–14].

To address the high global burden of *Shigella*, there are several *Shigella* vaccines in the pipeline, of which three are in Phase IIA and one in Phase III trials [15]. The World Health Organization (WHO) recently published preferred product characteristics (PPC) for a *Shigella* vaccine [1], and efforts are underway to define the full value proposition for such a vaccine. The primary goal of a *Shigella* vaccine is to prevent mortality and moderate-to-severe episodes of shigellosis with an efficacy target set by WHO of 60% or more. Assuming this target can be met in trials conducted in ideal settings, real-world estimates of the reduction in diarrhea episodes that would be expected after vaccine introduction are needed to predict population-level vaccine impact.

Furthermore, a *Shigella* vaccine may produce ancillary benefits that need to be quantified, specifically reductions in antibiotic exposures since diarrhea is a major cause of antibiotic use [8,9]. Vaccine impact on fluoroquinolone/macrolide (F/M) use is of particular interest as they are the recommended treatment by WHO for dysentery [11,16], and 34% of dysentery cases (12.8 episodes per 100 child-years) in children under 2 years of age in the MAL-ED birth cohort were attributed to *Shigella*. In addition to preventing exposures to antibiotics for *Shigella*, a *Shigella* vaccine could further reduce selective pressure on asymptomatic enteric pathogens (i.e., bystander pathogens) present in the gut at the time of shigellosis treatment. Bystander pathogens are not the target of treatment but nonetheless are still exposed to antibiotics and are therefore at risk for development of antimicrobial resistance (AMR). There were more than 7 antibiotic exposures per child-year for bystander enteropathogenic bacteria in MAL-ED [8].

To inform the vaccine value proposition, we aimed to quantify the potential impact of a *Shigella* vaccine on the incidence of *Shigella* diarrhea (severe and nonsevere), all diarrhea, and antibiotic use in the first 2 years of life via various potential vaccination strategies. We simulated vaccine introduction within the high-resolution data from the MAL-ED birth cohort study, which aimed to examine the effects of enteric infections and malnutrition on the health of children living in LMICs. The outcomes of interest were directly observed in this study, such that we did not need to make assumptions about the natural history of *Shigella*, other enteric pathogens, and antibiotic use. While a prior study estimated the global impact of a *Shigella* vaccine on diarrhea and stunting outcomes [17], we uniquely estimated the impact on antibiotic use outcomes and considered different vaccine efficacies and dosing schedules. We also quantified the potential impact of indirect protection for children who were too young to be vaccinated due to vaccination of vaccine-eligible children (i.e., herd immunity) and the impact of a vaccine that performs better for children who have been previously exposed to *Shigella*.

## Methods

### Study design and participants

The MAL-ED study design has been previously detailed [18]. Briefly, this study was conducted at 8 sites (Dhaka, Bangladesh; Fortaleza, Brazil; Vellore, India; Bhaktapur, Nepal; Naushero Feroze, Pakistan; Loreto, Peru; Venda, South Africa; and Haydom, Tanzania) from November 2009 to February 2014. Children were enrolled within 17 days of birth and followed for 2 years. Two times per week, fieldworkers conducted home visits to collect information on daily antibiotic use and presence of illness. Stool samples were collected monthly (nondiarrheal surveillance samples) and during diarrheal episodes. Diarrhea episodes were defined as 3 or more loose stools in a 24-hour period or the presence of blood in at least 1 stool. Diarrhea severity was determined by the modified Vesikari score, previously outlined [19].

### Stool testing

The QIAamp Fast DNA Stool Mini Kit (Qiagen) was used to extract total nucleic acid from the stool specimens [20]. To detect the presence of 29 enteropathogens via quantitative polymerase chain reaction (qPCR), TaqMan Array Cards (TAC) were run using AgPath One Step RT PCR kit (Thermo Fisher) [2]. The quantification cycle (Cq) to define pathogen detection was set to <35. *Shigella* spp. were detected by the *ipaH* gene, as previously outlined [2].

### Modeled vaccine impacts

We simulated the impact of vaccines on the following observed outcomes in the MAL-ED data. First, *Shigella* diarrhea was defined as diarrhea episodes with an episode-specific attributable fraction for *Shigella* (AF*e*) >0.5, regardless of other pathogens detected. AF*e*s were calculated as 1—(1/OR*e*), where OR*e* was the pathogen-specific and quantity-specific odds ratio (OR) from a generalized linear mixed model associating pathogen quantity with diarrhea [2]. Second, severe *Shigella* diarrhea was defined as *Shigella* diarrhea with a modified Vesikari score >6 [19]. Third, the number of severe diarrhea episodes of any etiology was defined as diarrhea due to any cause with a modified Vesikari score >6. Fourth, diarrhea episodes overall included any etiology (including episodes in which an infectious etiology was not identified) and severity.

For vaccine impacts on antibiotic use, we focused on F/Ms as specific drug classes of interest and additionally assessed any antibiotic use. Each diarrhea episode was considered treated with antibiotics if antibiotics were taken during any day of the illness episode. Antibiotic courses overall were defined by antibiotic courses given to the child for any reason, as previously determined [8]. Antibiotic courses were separated by 2 antibiotic-free days. Antibiotic exposures to bystander pathogens (i.e., pathogens present at the time of antibiotic treatment but that did not cause the illness that was treated) were defined by linking each antibiotic course to the most recent stool sample collected in the preceding 30 days. Any bacterial pathogens (atypical enteropathogenic *Escherichia coli* (*E*.*coli*), *Campylobacter*, enteroaggregative *E*. *coli*, enterotoxigenic *E*. *coli*, and typical enteropathogenic *E*. *coli*) detected in the linked stool were assumed to be bystander pathogens during the antibiotic course [8]. Antibiotic exposures to bystander pathogens were attributed to the treatment of *Shigella* if the antibiotic course was given during a diarrhea episode with a *Shigella* AFe >0.5.

### Vaccination scenarios

The characteristics of our simulated *Shigella* vaccine were modeled after those outlined in the WHO’s PPC for a *Shigella* vaccine [1] and those from vaccines currently in the pipeline [15]. We considered 1- and 2-dose *Shigella* vaccines with multiple potential vaccine dosing schedules that aligned with other existing vaccination events in the Expanded Programme on Immunizations (EPI) schedule [21]: a 1-dose vaccine with administration at 6 months or 9 months and 2-dose vaccines with administration at 6 and 9 months, 9 and 12 months, and 12 and 15 months (Table 1). Vaccine efficacy 14 days [22] after the second dose against severe *Shigella* diarrhea was simulated using a beta distribution with mean efficacy of 60% (Beta(α = 6, β = 4)) or 80% (Beta(α = 6, β = 1.5)) in separate scenarios. These beta distributions have 80% of values within an absolute 20% above and below the mean (e.g., 80% of values are between 40% and 80% for a mean vaccine efficacy of 60%), and our results were insensitive to changes to the assumed standard deviation of the beta distribution. 60% vaccine efficacy is the minimum preferred efficacy target outlined by the WHO’s PPC [1], and 80% vaccine efficacy scenario represents an optimistic scenario. Efficacy against nonsevere *Shigella* episodes was the simulated efficacy multiplied by 2/3 (for 60% mean severe vaccine efficacy) or 3/4 (for 80% mean severe vaccine efficacy) to target a mean efficacy against nonsevere episodes of 40% and 60%, respectively. Vaccine efficacy between the first dose up to 14 days after the second dose was half that which was applied 14 days after the second dose (Table 1).

**Table 1:** *Shigella* vaccination scenarios simulated in the MAL-ED dataset, including dosing schedules, efficacies, and inclusion of indirect and boosting protection [1].

Scenario	Dosing schedule	*Shigella* diarrhea severity	Mean VE* 14 days after first dose	Mean VE* 14 days after second dose	Indirect effect	Mean boosting effect after first dose, before second dose	Mean boosting effect after second dose
0	No vaccine	—	—	—	—	—	—
1	First: 6 months	Severe	60%	—	20%	+20% (80% VE)	—
		Non-severe	40%	—	20%	+20% (60% VE)	—
2	First: 9 months	Severe	60%	—	20%	+20% (80% VE)	—
		Non-severe	40%	—	20%	+20% (60% VE)	—
3	First: 6 months Second: 9 months	Severe	30%	60%	20%	+10% (40% VE)	+20% (80% VE)
		Non-severe	20%	40%	20%	+10% (30% VE)	+20% (60% VE)
4	First: 9 months Second: 12 months	Severe	30%	60%	20%	+10% (40% VE)	+20% (80% VE)
		Non-severe	20%	40%	20%	+10% (30% VE)	+20% (60% VE)
5	First: 12 months Second: 15 months	Severe	30%	60%	20%	+10% (40% VE)	+20% (80% VE)
		Non-severe	20%	40%	20%	+10% (30% VE)	+20% (60% VE)
6	First: 6 months	Severe	80%	—	20%	+20% (100% VE)	—
		Non-severe	60%	—	20%	+20% (80% VE)	—
7	First: 9 months	Severe	80%	—	20%	+20% (100% VE)	—
		Non-severe	60%	—	20%	20% (80% VE)	—
8	First: 6 months Second: 9 months	Severe	40%	80%	20%	+10% (50% VE)	+20% (100% VE)
		Non-severe	30%	60%	20%	+10% (40% VE)	+20% (80% VE)
9	First: 9 months Second: 12 months	Severe	40%	80%	20%	+10% (50% VE)	+20% (100% VE)
		Non-severe	30%	60%	20%	+10% (40% VE)	+20% (80% VE)
10	First: 12 months Second: 15 months	Severe	40%	80%	20%	+10% (50% VE)	+20% (100% VE)
		Non-severe	30%	60%	20%	+10% (40% VE)	+20% (80% VE)

VE, vaccine efficacy.

*60% vaccine efficacy for severe disease is the minimum preferred efficacy target outlined by the WHO’s PPC [1], and 80% vaccine efficacy scenario represents an optimistic scenario. VE for severe disease was randomly sampled from a beta distribution (Beta(α = 6, β = 4) for 60% mean efficacy and Beta(α = 6, β = 1.5) for 80% mean efficacy). VE against nonsevere *Shigella* episodes was this sampled efficacy multiplied by 2/3 (for 60% mean severe VE) or 3/4 (for 80% mean severe VE) to target a mean efficacy against nonsevere episodes of 40% and 60%, respectively. VE after the first dose was half that after the second dose.

For scenarios that assumed the vaccine would produce indirect protection for vaccine-ineligible children, we randomly selected 20% of *Shigella* diarrhea episodes that occurred in children under the age of the first dose of vaccine administration to be prevented. These simulated levels of indirect protection were based on what was observed with the Vi-tetanus toxoid conjugate vaccine in Bangladesh [23]. For instance, under a scenario with vaccine doses administered at 9 and 12 months, 20% of diarrhea episodes occurring in children under the age of 9 months were randomly prevented. For scenarios that assumed the vaccine would perform better among children previously exposed to *Shigella* (i.e., boosting protection), efficacy was increased by an absolute 20% at the mean for *Shigella* diarrhea episodes that occurred in children who had a *Shigella* infection prior to administration of the first dose of the vaccine. To do this, we multiplied the sampled efficacy for severe episodes by 4/3 and for nonsevere episodes by 3/2 for the 60% mean vaccine efficacy scenario. For the 80% mean vaccine efficacy scenario, we multiplied the sampled efficacy by 5/4 and 4/3 for severe and nonsevere episodes, respectively. For example, in the 9- and 12-month dosing vaccine scenario with 60% efficacy with 20% boosting effects, if a child was infected with *Shigella* prior to 9 months of age, mean efficacy went from 60% to 80%.

Results reported primarily in the text correspond to a vaccine with 2 doses at 9 and 12 months with a mean vaccine efficacy against severe episodes of 60% since these characteristics may be the most realistic among the range of acceptable parameters outlined in the WHO’s PPC [1]. Results from all other vaccination scenarios are described in the tables and figures.

### Statistical analysis

To simulate vaccine introduction within the observational MAL-ED data, we performed Monte Carlo simulations using random sampling with replacement of children to a sample size of 50,000. For each simulated vaccination scenario, we randomly selected observed *Shigella* diarrhea episodes from these children to be prevented by a probability equal to vaccine efficacy (sampled using a beta distribution as described above). In a no-vaccine scenario, no episodes were selected to be prevented. We then estimated the incidence of each diarrhea and antibiotic outcome defined above in the simulated scenario as the number with the outcome/person-time at risk, excluding the outcome episodes that were randomly selected to be prevented under each vaccination scenario. *Shigella*-specific incidence estimates were multiplied by the ratio of the total number of diarrhea episodes to the total number that were validly tested for *Shigella* by qPCR to account for episodes that did not have a stool sample collected and/or tested (ratio = 1.233). Bystander exposure incidence estimates were further multiplied by the ratio of the total number of antibiotic courses to the total number that could be linked to a stool sample collected in the preceding 30 days to extrapolate to courses that could not be linked (ratio = 1.152). Estimates and confidence intervals were estimated by the median, 2.5th and 97.5th percentiles of 1,000 iterations of this procedure. To quantify the expected reductions in the outcomes listed above, we estimated the absolute difference between each vaccine scenario and the no-vaccine scenario as the incidence rate difference $\left(|\;{Incidence}_{vaccine\;senario}-{Incidence}_{no\;vaccine\;senario}|\right)$, the relative difference as the incidence rate ratio $\left(\frac{{Incidence}_{vaccine\;senario}}{{Incidence}_{no\;vaccine\;senario}}\right)$, and the relative percent reduction $\left(\frac{{Incidence}_{no\;vaccine\;senario}-{Incidence}_{vaccine\;senario}}{{Incidence}_{no\;vaccine\;senario}}\right)$. We estimated each of these outcomes overall and by sites to investigate heterogeneity of impact by site. The statistical analysis plan is available in S1 Protocol.

All statistical analyses were performed via R software, version 4.0.2 (Foundation for Statistical Computing).

### Ethics approvals

This study involves human participants. For the parent study, ethical approval was obtained from the Institutional Review Boards at the University of Virginia School of Medicine (Charlottesville, USA) (14595) and at each of the participating research sites: Ethical Review Committee, ICDDR,B (Bangladesh); Committee for Ethics in Research, Universidade Federal do Ceara; National Ethical Research Committee, Health Ministry, Council of National Health (Brazil); Institutional Review Board, Christian Medical College, Vellore; Health Ministry Screening Committee, Indian Council of Medical Research (India); Institutional Review Board, Institute of Medicine, Tribhuvan University; Ethical Review Board, Nepal Health Research Council; Institutional Review Board, Walter Reed Army Institute of Research (Nepal); Institutional Review Board, Johns Hopkins University; PRISMA Ethics Committee; Health Ministry, Loreto (Peru); Ethical Review Committee, Aga Khan University (Pakistan); Health, Safety and Research Ethics Committee, University of Venda; Department of Health and Social Development, Limpopo Provincial Government (South Africa); Medical Research Coordinating Committee, National Institute for Medical Research; Chief Medical Officer, Ministry of Health and Social Welfare (Tanzania). For the current study, we obtained ethical approval at the University of Virginia School of Medicine (Charlottesville, USA) (22398) and Emory University (Atlanta, USA) (STUDY00003285). Caregivers provided written informed consent for their child to participate in the study before taking part.

## Results

These analyses included 1,715 children, of which 83% (*n* = 1,427) had at least 1 *Shigella* infection during their first 2 years of life (Table 2). There were 273.8 diarrhea episodes of any severity per 100 child-years (*n* = 9,392) and 30.6 *Shigella* diarrhea episodes per 100 child-years (*n* = 754). Caregivers reported 457.6 courses per 100 child-years of antibiotics (*n* = 15,697), among which 110.1 courses per 100 child-years (*n* = 3,775) were to treat diarrhea episodes of any etiology and of which 16.3 courses per 100 child-years were attributable to *Shigella* diarrhea (*n* = 427). Bystander pathogens had 744.1 (*n* = 22,161) and 32.9 (*n* = 750) exposures to antibiotics per 100 child-years resulting from any antibiotic use and resulting from the treatment of *Shigella*, respectively.

**Table 2:** Diarrhea episodes, antibiotic use, and bystander pathogen exposures to antibiotics among 1,715 children enrolled in the MAL-ED cohort.

	<6 months	≥6 months, <9 months	≥9 months, <12 months	≥12 months, <15 months	≥15 months
No. children with their first instance of a *Shigella* infection, n (%)^a^^,^^d^	163 (9.5)	199 (11.6)	275 (16.0)	251 (14.6)	539 (31.4)
No. severe *Shigella* diarrhea episodes, n (rate)^b^^,^^c^^,^^d^	5 (0.8)	9 (2.9)	10 (3.2)	17 (5.5)	48 (5.2)
No. severe diarrhea episodes of any etiology, n (rate)^b^^,^^c^	434 (50.6)	286 (66.7)	192 (44.8)	186 (43.4)	290 (22.5)
No. *Shigella* diarrhea episodes, n (rate)^b^^,^^c^^,^^d^	16 (2.6)	43 (14.0)	84 (27.3)	118 (38.3)	493 (53.3)
No. diarrhea episodes of any etiology, n (rate)^b^^,^^c^	2,386 (278.3)	1,498 (349.4)	1,333 (310.9)	1,236 (288.3)	2,939 (228.5)
No. antibiotic courses for severe *Shigella* diarrhea episodes, n (rate)^b^^,^^d^	6 (0.9)	6 (1.8)	12 (3.7)	12 (3.7)	32 (3.3)
No. antibiotic courses for severe diarrhea episodes of any etiology, n (rate)^b^	253 (29.5)	206 (48)	145 (33.8)	103 (24.0)	184 (14.3)
No. antibiotic courses for *Shigella* diarrhea episodes, n (rate)^b^^,^^d^	11 (1.7)	24 (7.4)	53 (16.3)	73 (22.4)	266 (27.3)
No. antibiotic courses for diarrhea episodes of any etiology, n (rate)^b^	804 (93.8)	629 (146.7)	588 (137.1)	480 (112.0)	1,274 (99.0)
No. antibiotic courses overall, n (rate)^b^	3,478 (405.6)	2,283 (532.5)	2,164 (504.7)	2,105 (491.0)	5,667 (440.6)
No. antibiotic exposures to bystander pathogens due to *Shigella* treatment, n (rate)^b^^,^^d^	25 (4.4)	39 (13.8)	102 (36.1)	133 (47.1)	451 (53.2)
No. antibiotic exposures to bystander pathogens overall, n (rate)^b^	2,736 (367.5)	3,404 (914.4)	3,641 (978.1)	3,527 (947.4)	8,853 (792.7)

^**a**^Denominator = 1,715 kids.

^**b**^Rate is per 100 child-years.

^**c**^Includes episodes that were and were not treated by antibiotics.

^**d**^Counted among infections/episodes/exposures in which stools were collected with valid qPCR test results for *Shigella*.

Rates are extrapolated to all infections/episodes/exposures.

### Prevention of diarrhea

**Fig 1:**
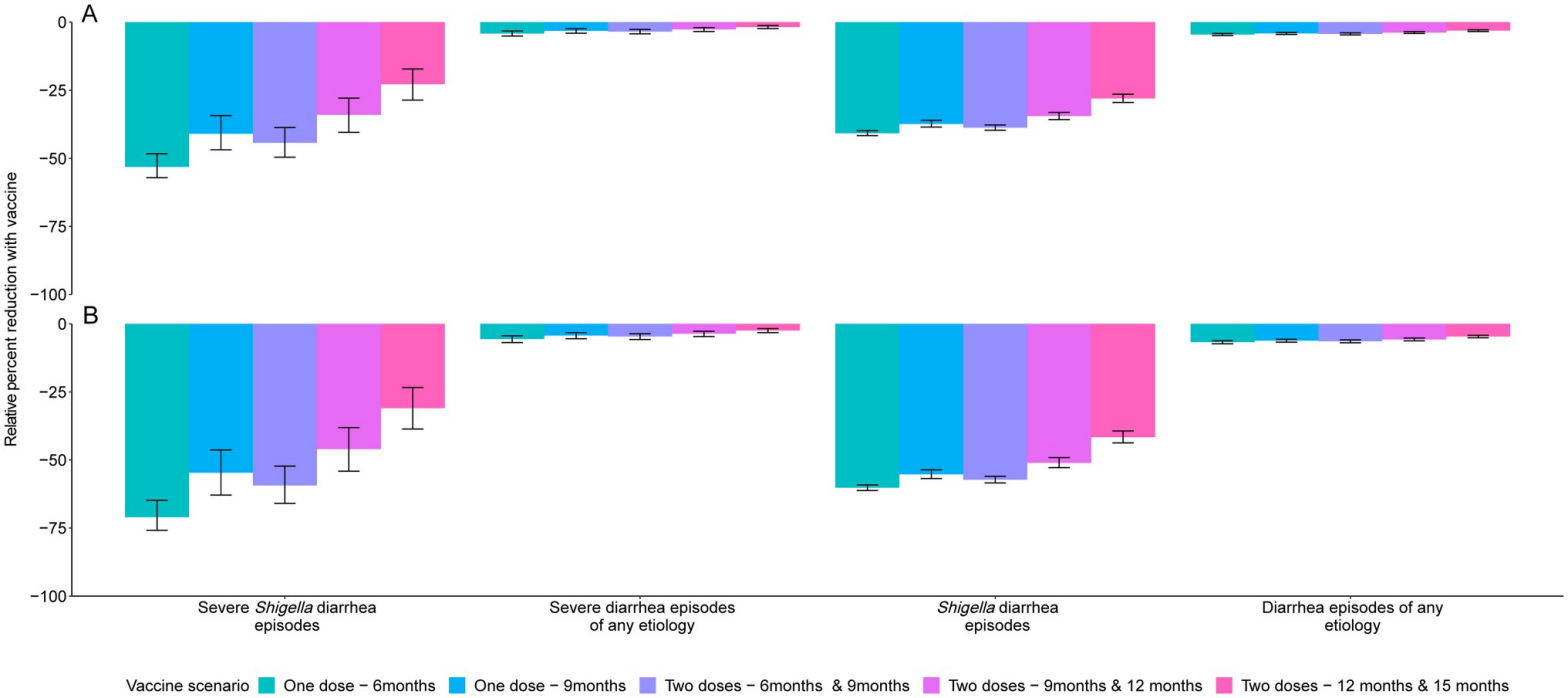
Relative percent reductions in diarrhea outcomes for 5 *Shigella* vaccine scenarios with 60% (A) and 80% (B) full vaccine efficacies against severe *Shigella* diarrhea and no indirect or boosting protection. Caption: The black lines represent 95% confidence intervals.

A *Shigella* vaccine given at 9 and 12 months with a mean 60% vaccine efficacy would be expected to prevent 1.2 (95% CI [0.9, 1.6]) severe *Shigella* diarrhea episodes and 10.6 (95% CI [9.5, 11.5]) *Shigella* diarrhea episodes of any severity per 100 child-years (Table 3), which corresponds to a relative 34.0% reduction in severe shigellosis episodes and a relative 34.5% reduction in shigellosis episodes of any severity (Fig 1, Table A in S1 Appendix). While the vaccine would reduce the same number of severe and all diarrhea episodes due to any etiology, the relative percent reductions would be smaller, at 2.7% for severe diarrhea episodes of any etiology and 3.8% for diarrhea episodes of any etiology (Table 3, Fig 1, Table A in S1 Appendix).

**Table 3:** Absolute (incidence rate differences) and relative (incidence rate ratios) differences in diarrhea outcomes for 5 *Shigella* vaccine scenarios compared to the no-vaccine scenario with 60% and 80% full vaccine efficacies against severe *Shigella* diarrhea and no indirect or boosting protection.

Vaccine scenario and efficacy outcome	Incidence rate difference (cases per 100 child-years)		Incidence rate ratio	
	60% VE (95% CI)	80% VE (95% CI)	60% VE (95% CI)	80% VE (95% CI)
One dose—6 months				
Severe *Shigella* diarrhea episodes	−1.9 (−2.4, −1.5)	−2.6 (−3.2, −2.0)	0.47 (0.43, 0.52)	0.29 (0.24, 0.35)
Severe diarrhea episodes of any etiology	−1.9 (−2.4, −1.5)	−2.6 (−3.2, −2.0)	0.96 (0.95, 0.97)	0.94 (0.93, 0.96)
*Shigella* diarrhea episodes	−12.5 (−13.6, −11.4)	−18.5 (−20.1, −16.8)	0.59 (0.58, 0.60)	0.40 (0.39, 0.41)
Diarrhea episodes of any etiology	−12.5 (−13.6, −11.4)	−18.5 (−20.1, −16.8)	0.95 (0.95, 0.96)	0.93 (0.93, 0.94)
One dose—9 months				
Severe *Shigella* diarrhea episodes	−1.5 (−1.9, −1.1)	−2.0 (−2.5, −1.5)	0.59 (0.53, 0.66)	0.45 (0.37, 0.54)
Severe diarrhea episodes of any etiology	−1.5 (−1.9, −1.1)	−2.0 (−2.5, −1.5)	0.97 (0.96, 0.98)	0.96 (0.95, 0.97)
*Shigella* diarrhea episodes	−11.5 (−12.5, −10.4)	−16.9 (−18.5, −15.4)	0.63 (0.61, 0.64)	0.45 (0.43, 0.46)
Diarrhea episodes of any etiology	−11.5 (−12.5, −10.4)	−16.9 (−18.5, −15.4)	0.96 (0.95, 0.96)	0.94 (0.93, 0.94)
Two doses—6 months and 9 months				
Severe *Shigella* diarrhea episodes	−1.6 (−2.0, −1.2)	−2.1 (−2.7, −1.7)	0.56 (0.50, 0.61)	0.41 (0.34, 0.48)
Severe diarrhea episodes of any etiology	−1.6 (−2.0, −1.2)	−2.1 (−2.7, −1.7)	0.97 (0.96, 0.97)	0.95 (0.94, 0.96)
*Shigella* diarrhea episodes	−11.9 (−12.9, −10.8)	−17.6 (−19.1, −15.9)	0.61 (0.60, 0.62)	0.43 (0.42, 0.44)
Diarrhea episodes of any etiology	−11.9 (−12.9, −10.8)	−17.6 (−19.1, −15.9)	0.96 (0.95, 0.96)	0.94 (0.93, 0.94)
Two doses—9 months and 12 months				
Severe *Shigella* diarrhea episodes	−1.2 (−1.6, −0.9)	−1.7 (−2.1, −1.2)	0.66 (0.60, 0.72)	0.54 (0.46, 0.62)
Severe diarrhea episodes of any etiology	−1.2 (−1.6, −0.9)	−1.7 (−2.1, −1.2)	0.97 (0.97, 0.98)	0.96 (0.95, 0.97)
*Shigella* diarrhea episodes	−10.6 (−11.5, −9.5)	−15.6 (−17.1, −14.1)	0.66 (0.64, 0.67)	0.49 (0.47, 0.51)
Diarrhea episodes of any etiology	−10.6 (−11.5, −9.5)	−15.6 (−17.1, −14.1)	0.96 (0.96, 0.97)	0.94 (0.94, 0.95)
Two doses—12 months and 15 months				
Severe *Shigella* diarrhea episodes	−0.8 (−1.1, −0.6)	−1.1 (−1.5, −0.8)	0.77 (0.71, 0.83)	0.69 (0.61, 0.77)
Severe diarrhea episodes of any etiology	−0.8 (−1.1, −0.6)	−1.1 (−1.5, −0.8)	0.98 (0.98, 0.99)	0.98 (0.97, 0.98)
*Shigella* diarrhea episodes	−8.6 (−9.5, −7.7)	−12.7 (−14.0, −11.4)	0.72 (0.71, 0.74)	0.58 (0.56, 0.61)
Diarrhea episodes of any etiology	−8.6 (−9.5, −7.7)	−12.7 (−14.0, −11.4)	0.97 (0.97, 0.97)	0.95 (0.95, 0.96)

CI, confidence interval; VE, vaccine efficacy.

**Fig 2:**
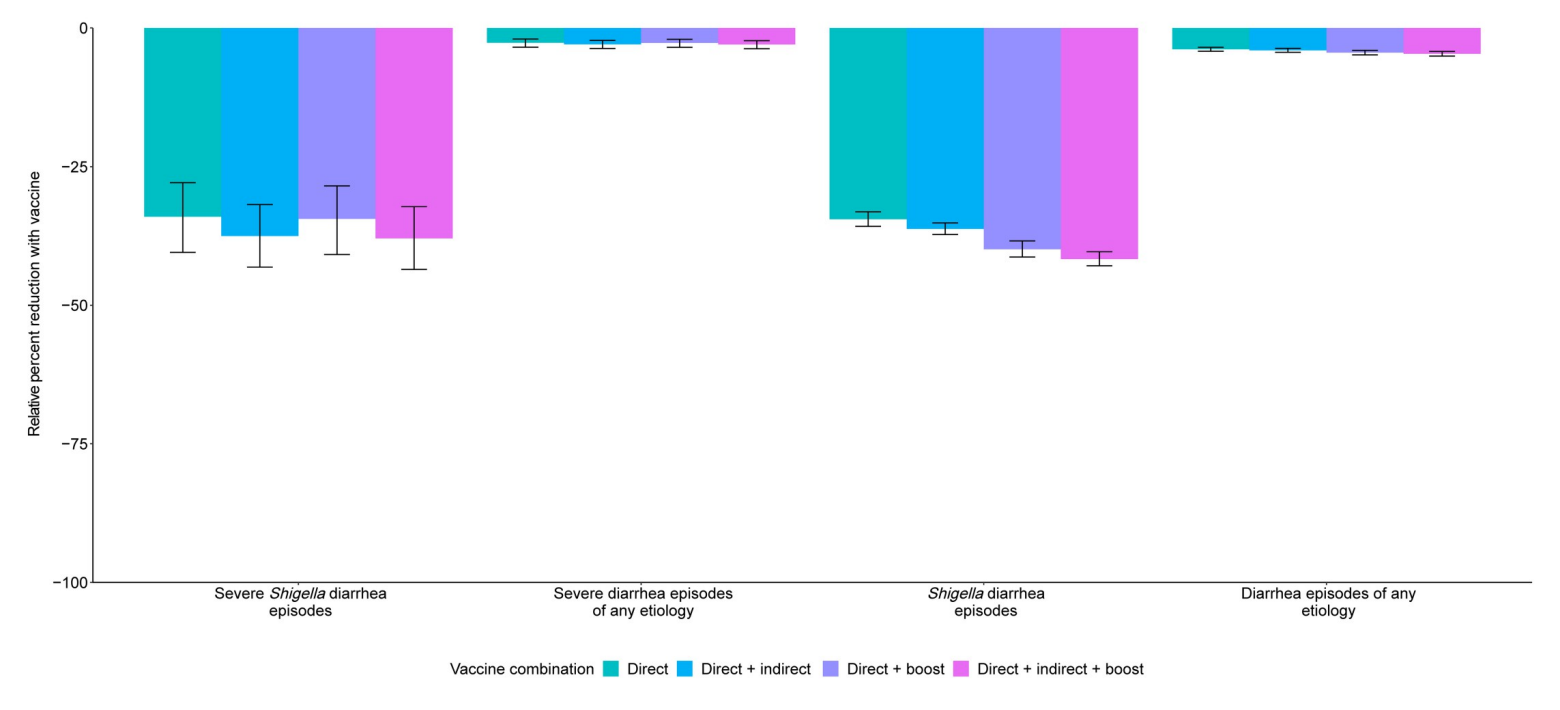
Relative percent reductions in diarrhea outcomes with the addition of indirect and boosting protection for the 9- and 12-month *Shigella* vaccine dosing scenario with 60% full vaccine efficacy against severe *Shigella* diarrhea. Caption: The black lines represent 95% confidence intervals.

The 10.6 (95% CI [9.5, 11.5]) prevented *Shigella* diarrhea episodes per 100 child-years increased slightly to 11.1 (95% CI [10.0, 12.1]) prevented *Shigella* diarrhea episodes per 100 child-years when analyses further allowed for 20% indirect protection (Table B in S1 Appendix). This same vaccine with 20% boosting protection and no indirect protection would prevent 12.2 (95% CI [11.0, 13.4]) *Shigella* diarrhea episodes per 100 child-years (Table C in S1 Appendix). Together, a vaccine with direct effects plus indirect and boosting protection effects would prevent 12.8 (95% CI [11.6, 14.0]) *Shigella* diarrhea episodes per 100 child-years (Table D in S1 Appendix), which equates to a relative 37.9% reduction in severe *Shigella* diarrhea episodes and a relative 41.7% reduction in *Shigella* diarrhea episodes (Fig 2, Table E in S1 Appendix).

### Prevention of antibiotic use

**Fig 3:**
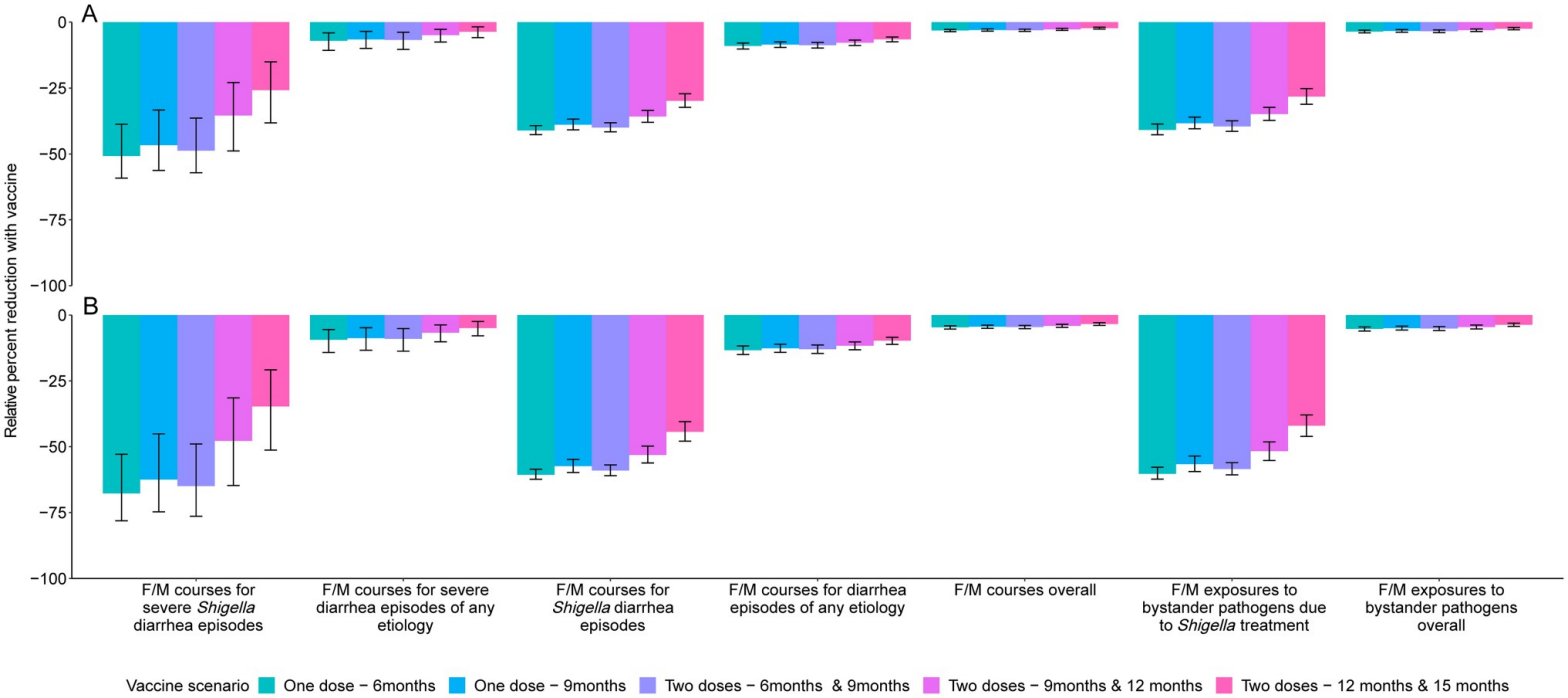
Relative percent reductions in fluroquinolone and macrolide (F/M) use outcomes for 5 *Shigella* vaccine scenarios with 60% (A) and 80% (B) full vaccine efficacies against severe *Shigella* diarrhea and no indirect or boosting protection. Caption: The black lines represent 95% confidence intervals.

**Fig 4:**
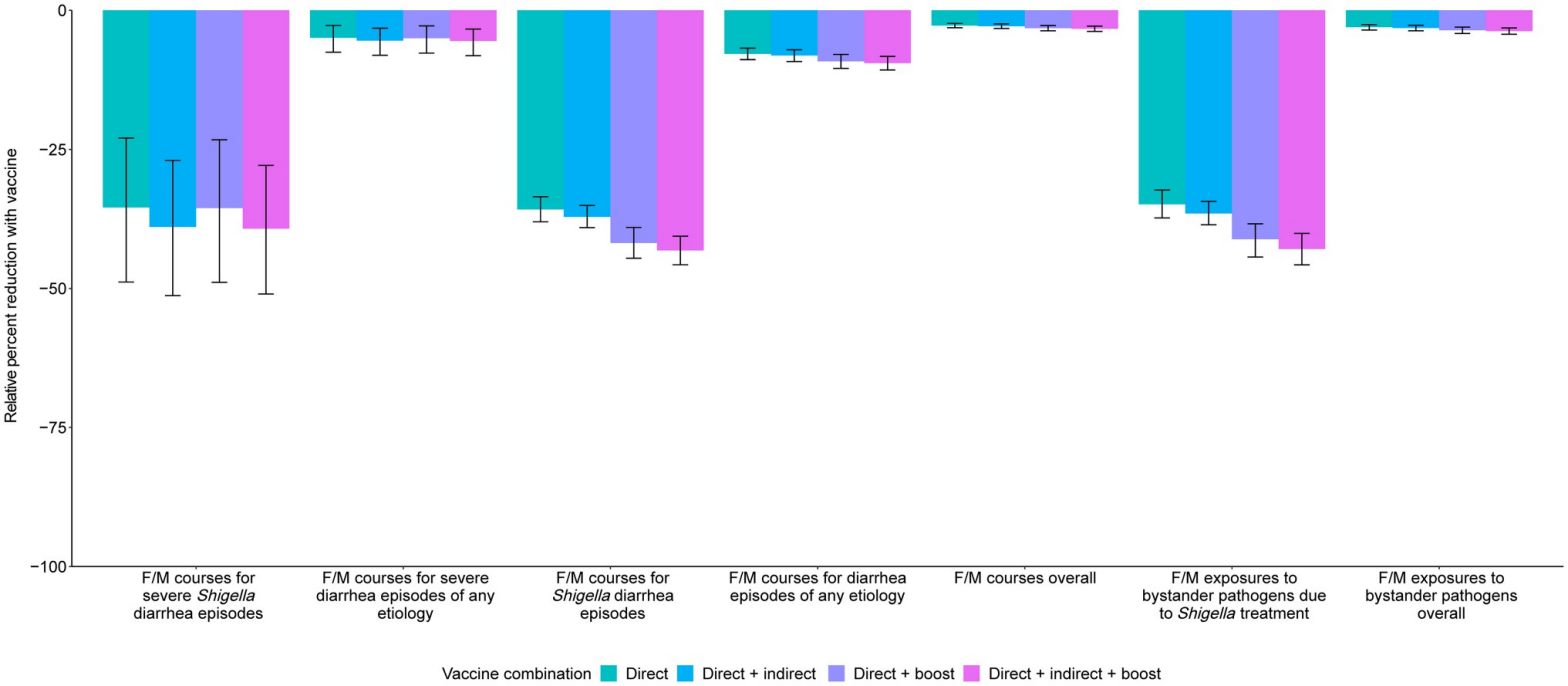
Relative percent reductions in fluroquinolone and macrolide (F/M) use outcomes with the addition of indirect and boosting protection for the 9- and 12-month *Shigella* vaccine dosing scenario with 60% full vaccine efficacy against severe *Shigella* diarrhea. Caption: The black lines represent 95% confidence intervals.

A 2-dose *Shigella* vaccine given at 9 and 12 months with a mean 60% vaccine efficacy would prevent 0.3 (95% CI [0.2, 0.5]) F/M courses for severe *Shigella* diarrhea episodes (relative 35.4% reduction), 3.0 (95% CI [2.5, 3.5]) F/M courses for *Shigella* episodes (relative 35.8% reduction), and 6.1 (95% CI [5.0, 7.3]) F/M exposures to bystander pathogens due to *Shigella* treatment (relative 34.9% reduction) per 100 child-years (Table 4, Fig 3, Table F in S1 Appendix). However, this vaccine would reduce 3.0 (95% CI [2.5, 3.5]) overall F/M courses and 6.1 (95% CI [5.0, 7.3]) overall F/M exposures to bystander pathogens per 100 child-years by only 2.8% and 3.1%, respectively (Table F in S1 Appendix). The 3.0 (95% CI [2.5, 3.5]) prevented F/M courses for *Shigella* diarrhea episodes per 100 child-years increased to 3.1 (95% CI [2.6, 3.6]) prevented episodes with added indirect protection effects (Table G in S1 Appendix), to 3.5 (95% CI [2.9, 4.1]) with added boosting effects (Table H in S1 Appendix), and to 3.6 (95% CI [3.0, 4.2]) when both indirect effects and boosting effects were added to the direct effects (Table I in S1 Appendix) per 100 child-years. When indirect and boosting effects were added to the direct effects, there were slight increases in relative percent reductions of all metrics: F/M courses for *Shigella* diarrhea episodes (35.8% to 43.2%), F/M courses overall (2.8% to 3.3%), F/M exposures to bystander pathogens due to *Shigella* treatment (34.9% to 42.9%), and F/M exposures to bystander pathogens overall (3.1% to 3.7%) (Fig 4, Table J in S1 Appendix).

**Table 4:** Absolute (incidence rate differences) and relative (incidence rate ratios) differences in fluoroquinolone/macrolide (F/M) outcomes for 5 *Shigella* vaccine scenarios compared to the no-vaccine scenario with 60% and 80% full vaccine efficacies against severe *Shigella* diarrhea and no indirect or boosting protection.

Vaccine scenario and efficacy outcome	Incidence rate difference (cases per 100 child-years)		Incidence rate ratio	
	60% VE (95% CI)	80% VE (95% CI)	60% VE (95% CI)	80% VE (95% CI)
One dose—6 months				
F/M courses for severe *Shigella* diarrhea episodes	−0.5 (−0.7, −0.3)	−0.6 (−1.0, −0.4)	0.49 (0.41, 0.61)	0.32 (0.22, 0.47)
F/M courses for severe diarrhea episodes of any etiology	−0.5 (−0.7, −0.3)	−0.6 (−1.0, −0.4)	0.93 (0.89, 0.96)	0.91 (0.86, 0.94)
F/M courses for *Shigella* diarrhea episodes	−3.4 (−4.0, −2.9)	−5.0 (−5.9, −4.2)	0.59 (0.57, 0.61)	0.39 (0.38, 0.41)
F/M courses for diarrhea episodes of any etiology	−3.4 (−4.0, −2.9)	−5.0 (−5.9, −4.2)	0.91 (0.90, 0.92)	0.87 (0.85, 0.88)
F/M courses overall	−3.4 (−4.0, −2.9)	−5.0 (−5.9, −4.2)	0.97 (0.96, 0.97)	0.95 (0.95, 0.96)
F/M exposures to bystander pathogens due to *Shigella* treatment	−7.1 (−8.5, −5.9)	−10.5 (−12.5, −8.7)	0.59 (0.57, 0.61)	0.40 (0.38, 0.42)
F/M exposures to bystander pathogens overall	−7.1 (−8.5, −5.9)	−10.5 (−12.5, −8.7)	0.96 (0.96, 0.97)	0.95 (0.94, 0.95)
One dose—9 months				
F/M courses for severe *Shigella* diarrhea episodes	−0.4 (−0.7, −0.2)	−0.6 (−0.9, −0.3)	0.53 (0.44, 0.67)	0.38 (0.25, 0.55)
F/M courses for severe diarrhea episodes of any etiology	−0.4 (−0.7, −0.2)	−0.6 (−0.9, −0.3)	0.93 (0.90, 0.96)	0.91 (0.87, 0.95)
F/M courses for *Shigella* diarrhea episodes	−3.2 (−3.8, −2.7)	−4.8 (−5.6, −4.0)	0.61 (0.59, 0.63)	0.43 (0.40, 0.45)
F/M courses for diarrhea episodes of any etiology	−3.2 (−3.8, −2.7)	−4.8 (−5.6, −4.0)	0.91 (0.90, 0.92)	0.87 (0.86, 0.89)
F/M courses overall	−3.2 (−3.8, −2.7)	−4.8 (−5.6, −4.0)	0.97 (0.97, 0.97)	0.96 (0.95, 0.96)
F/M exposures to bystander pathogens due to *Shigella* treatment	−6.7 (−8.0, −5.5)	−9.9 (−11.8, −8.1)	0.62 (0.60, 0.64)	0.43 (0.41, 0.46)
F/M exposures to bystander pathogens overall	−6.7 (−8.0, −5.5)	−9.9 (−11.8, −8.1)	0.97 (0.96, 0.97)	0.95 (0.94, 0.96)
Two doses—6 months and 9 months				
F/M courses for severe *Shigella* diarrhea episodes	−0.5 (−0.7, −0.2)	−0.6 (−1.0, −0.3)	0.51 (0.43, 0.64)	0.35 (0.24, 0.51)
F/M courses for severe diarrhea episodes of any etiology	−0.5 (−0.7, −0.2)	−0.6 (−1.0, −0.3)	0.93 (0.90, 0.96)	0.91 (0.86, 0.95)
F/M courses for *Shigella* diarrhea episodes	−3.3 (−3.9, −2.8)	−4.9 (−5.8, −4.1)	0.60 (0.58, 0.62)	0.41 (0.39, 0.43)
F/M courses for diarrhea episodes of any etiology	−3.3 (−3.9, −2.8)	−4.9 (−5.8, −4.1)	0.91 (0.90, 0.92)	0.87 (0.85, 0.89)
F/M courses overall	−3.3 (−3.9, −2.8)	−4.9 (−5.8, −4.1)	0.97 (0.96, 0.97)	0.95 (0.95, 0.96)
F/M exposures to bystander pathogens due to *Shigella* treatment	−6.9 (−8.2, −5.7)	−10.2 (−12.2, −8.4)	0.60 (0.59, 0.63)	0.42 (0.39, 0.44)
F/M exposures to bystander pathogens overall	−6.9 (−8.2, −5.7)	−10.2 (−12.2, −8.4)	0.97 (0.96, 0.97)	0.95 (0.94, 0.96)
Two doses—9 months and 12 months				
F/M courses for severe *Shigella* diarrhea episodes	−0.3 (−0.5, −0.2)	−0.5 (−0.7, −0.2)	0.65 (0.51, 0.77)	0.52 (0.35, 0.69)
F/M courses for severe diarrhea episodes of any etiology	−0.3 (−0.5, −0.2)	−0.5 (−0.7, −0.2)	0.95 (0.92, 0.97)	0.93 (0.90, 0.96)
F/M courses for *Shigella* diarrhea episodes	−3.0 (−3.5, −2.5)	−4.4 (−5.2, −3.7)	0.64 (0.62, 0.67)	0.47 (0.44, 0.50)
F/M courses for diarrhea episodes of any etiology	−3.0 (−3.5, −2.5)	−4.4 (−5.2, −3.7)	0.92 (0.91, 0.93)	0.88 (0.87, 0.90)
F/M courses overall	−3.0 (−3.5, −2.5)	−4.4 (−5.2, −3.7)	0.97 (0.97, 0.98)	0.96 (0.95, 0.97)
F/M exposures to bystander pathogens due to *Shigella* treatment	−6.1 (−7.3, −5.0)	−9.1 (−10.9, −7.4)	0.65 (0.63, 0.68)	0.48 (0.45, 0.52)
F/M exposures to bystander pathogens overall	−6.1 (−7.3, −5.0)	−9.1 (−10.9, −7.4)	0.97 (0.96, 0.97)	0.95 (0.95, 0.96)
Two doses—12 months and 15 months				
F/M courses for severe *Shigella* diarrhea episodes	−0.2 (−0.4, −0.1)	−0.3 (−0.6, −0.2)	0.74 (0.62, 0.85)	0.65 (0.49, 0.79)
F/M courses for severe diarrhea episodes of any etiology	−0.2 (−0.4, −0.1)	−0.3 (−0.6, −0.2)	0.96 (0.94, 0.98)	0.95 (0.92, 0.98)
F/M courses for *Shigella* diarrhea episodes	−2.5 (−2.9, −2.0)	−3.7 (−4.4, −3.0)	0.70 (0.68, 0.73)	0.56 (0.52, 0.60)
F/M courses for diarrhea episodes of any etiology	−2.5 (−2.9, −2.0)	−3.7 (−4.4, −3.0)	0.93 (0.93, 0.94)	0.90 (0.89, 0.92)
F/M courses overall	−2.5 (−2.9, −2.0)	−3.7 (−4.4, −3.0)	0.98 (0.97, 0.98)	0.97 (0.96, 0.97)
F/M exposures to bystander pathogens due to *Shigella* treatment	−4.9 (−6.0, −3.9)	−7.4 (−8.9, −5.9)	0.72 (0.69, 0.75)	0.58 (0.54, 0.62)
F/M exposures to bystander pathogens overall	−4.9 (−6.0, −3.9)	−7.4 (−8.9, −5.9)	0.98 (0.97, 0.98)	0.96 (0.96, 0.97)

CI, confidence interval; F/M, fluoroquinolone/macrolide; VE, vaccine efficacy.

While a 2-dose *Shigella* vaccine given at 9 and 12 months with a mean 60% vaccine efficacy would prevent more instances of antibiotic use overall than of F/M specifically, the percent reductions in overall antibiotic use were smaller than what was observed with F/M use (Table K in S1 Appendix, Fig A in S1 Appendix, Table L in S1 Appendix). In this scenario, 0.9 (95% CI [0.6, 1.2]) antibiotic courses for severe *Shigella* diarrhea episodes (relative 33.5% reduction), 5.6 (95% CI [5.0, 6.3]) antibiotic courses for *Shigella* diarrhea episodes (relative 34.5% reduction), and 10.9 (95% CI [9.5, 12.5]) antibiotic exposures to bystander pathogens due to *Shigella* treatment (relative 33.9% reduction) per 100 child-years were prevented (Table K in S1 Appendix, Fig A in S1 Appendix, Table L in S1 Appendix). However, there was only a relative 0.9% (−5.6 antibiotic courses per 100 child-years; 95% CI [−6.3, −5.0]) and relative 1.1% (−10.9 antibiotic courses per 100 child-years; 95% CI [−12.5, −9.5]) reduction in overall antibiotic use and overall exposures to bystander pathogens, respectively (Table L in S1 Appendix). Similar to what was observed with F/M, the addition of indirect and boosting effects onto the direct effects minimally increased the number of prevented outcomes (Tables M-P in S1 Appendix, Fig B in S1 Appendix).

There was substantial variability in vaccine impact by site. The greatest absolute reductions (incidence rate differences) in diarrhea episodes and antibiotic use were observed in the Bangladesh and Peru sites, and less impact was observed in the Brazil, South Africa, and Tanzania sites due to lower burden of *Shigella* diarrhea in these sites (Tables Q-S in S1 Appendix; Fig C in S1 Appendix). F/M use for *Shigella* treatment was also variable across sites resulting in little to no impact of a *Shigella* vaccine on F/M use in the Brazil, Pakistan, South Africa, and Tanzania sites (Fig D in S1 Appendix). The vaccine would be expected to prevent 2.0 (95% CI [1.6, 2.4]) F/M courses per 100 child-years in Bangladesh, and 0.7 (95% CI [0.5, 0.9]), 0.1 (95% CI [0.0, 0.2]), and 0.1 (95% CI [0.1, 0.3]) F/M courses per 100-child years in Peru, India, and Nepal, respectively.

The corresponding results for the other vaccine scenarios listed in Table 1 are displayed in Figs 1 and 2, Tables 3 and 4, Fig B in S1 Appendix, Tables A-D, F-I, and K-O in S1 Appendix). In general, the earlier the vaccine is given, the greater the expected reduction in diarrhea episodes and antibiotic use. Additionally, the single-dose vaccines were more efficacious than the 2-dose vaccines initiated at the same time (e.g., 1 dose at 9 months versus 2 doses at 9 and 12 months) since the full efficacy was achieved at an earlier age with the single-dose vaccines.

## Discussion

A *Shigella* vaccine administered at 9 and 12 months with a mean 60% vaccine efficacy could provide a substantial reduction in severe *Shigella* diarrhea episodes, *Shigella* diarrhea episodes of any severity, and F/M courses for *Shigella* diarrhea episodes. However, given the multitude of causes of diarrhea and antibiotic use in this population, the expected reductions in all-cause diarrhea and antibiotic use overall were modest (<5%). While single-dose vaccines and vaccines given at younger ages would prevent more diarrhea and antibiotic use, none of the vaccine candidates in clinical development meet those criteria. The vaccine schedules starting later in infancy, and particularly the schedule starting at 12 months, prevent substantially less disease and antibiotic use than the earlier schedules, supporting the preference in the WHO PPC for the vaccine schedule to be completed by 12 months of age [1]. This pattern reflects the increasing incidence of *Shigella* diarrhea in the first year of life, which nearly doubled from 9 to 12 months of age compared to 6 to 9 months. Because severe disease is more common in younger children, the expected relative reductions in severe outcomes were particularly less than those for nonsevere outcomes for vaccine strategies with older ages of administration. The primary driver of differences in results across simulations was the assumed vaccine efficacy followed by the vaccine dosing schedule. Incorporation of indirect protection and boosting only slightly increased the number of diarrhea episodes and antibiotic courses expected to be preventable, suggesting that these nuances will not be major determinants of vaccine success.

Our study builds on a recent study that modeled the global impact of *Shigella* vaccines [17] by considering a broader range of vaccine assumptions and scenarios, including variations in efficacy and dosing schedules, and impacts of partial protection after a first dose and herd immunity. Despite these differences, the studies estimated similar absolute reductions in *Shigella* diarrhea episodes (approximately 7 episodes per 100 child-years [17] compared to approximately 11 episodes per 100 child-years in our study). The difference in geographic scope between the 2 analyses resulting in different *Shigella* diarrhea incidence rates is a likely major contributor to the differences between the results. Uniquely, we also estimated the effects of *Shigella* vaccines on antibiotic use.

Our simulation approach took advantage of the high-resolution data available from the MAL-ED birth cohort such that we did not need to make assumptions about the natural history of *Shigella*, other enteric pathogens, and antibiotic use. We instead only made assumptions about the potential *Shigella* vaccines (e.g., dosing schedules, efficacy) and simulated their introduction within the observed data. While this approach is likely to provide accurate estimates of vaccine impact at the MAL-ED sites, the results may have limited generalizability to settings that are not comparable. The antibiotic use results may also have limited generalizability if antibiotic use practices change substantially over time. Because the MAL-ED study was conducted in 8 diverse sites across 3 continents, we are confident that our study provides broadly generalizable inferences to similar low-resource settings.

There were several limitations to our analysis. First, our analysis assumed that vaccine efficacy varies randomly across the population. However, it may be that the level of protection is dependent on vaccine-related factors or host characteristics such as age and malnutrition status. These factors could be incorporated into future analyses once they are better characterized for the *Shigella* vaccines in development. Next, a *Shigella* vaccine will likely not be protective against all serotypes, but given the lack of serotyping data, we were unable to simulate the prevention of episodes at the serotype level. Our estimates assume 100% cross protection for subtypes not included in the vaccine and therefore may be slightly overestimated depending on the true levels of cross protection. Third, while our results estimate the upper limit of the potential benefit of a *Shigella* vaccine since we assumed 100% vaccine coverage, it is likely that vaccine coverage would be lower in a real-world setting. Finally, because we only had diagnostic testing for enteric pathogens, we were unable to estimate bystander antibiotic exposure effects on subclinically carried respiratory pathogens and the larger microbiome, which may also have implications for antimicrobial resistance.

Estimation of *Shigella* vaccine impact could be extended in several ways. First, we did not consider the potential for waning immunity since we only observed outcomes to 2 years of age. The effects of waning would likely occur more than 6 months after the last vaccine dose, which was outside of our follow-up period for most vaccine scenarios. However, if efficacy wanes substantially before 2 years of age, our expected reductions may be overestimated. Next, we did not consider a 3-dose vaccine despite there being several 3-dose *Shigella* vaccines in the development pipeline [24–27]. Our estimates of the expected reductions in outcomes would apply to a 3-dose vaccine if the full efficacy is achieved after 2 doses and may be overestimates if full efficacy is not achieved until a third dose. Finally, our predicted reductions in antibiotic use could be underestimates if suspicion of *Shigella* is the main reason for treating diarrhea regardless of etiology, such that treatment rates also decline for other diarrhea etiologies after *Shigella* incidence is known to have been substantially reduced by the vaccine. This downstream impact of a *Shigella* vaccine could also be predicted.

Given the high burden of shigellosis and antibiotic treatment of shigellosis, a *Shigella* vaccine could make a substantial impact on *Shigella* burden, in terms of absolute reduction in diarrhea episodes, and have ancillary benefits in the reduction of antibiotic use. The observed heterogeneity in vaccine impact by site suggests that local data on *Shigella* incidence will be important for policymakers’ decisions about whether to introduce a *Shigella* vaccine. The absolute reductions in F/M use achieved by a *Shigella* vaccine accounted for roughly half the achievable reduction of all antibiotic use. However, there were greater relative reductions in F/M use compared to all antibiotic use since F/Ms are often targeted for diarrhea treatment and specifically for dysentery presumed to be shigellosis. F/M use has been associated with resistance in these drug classes [8,10,28], suggesting that reductions in use achievable by a *Shigella* vaccine could limit drug-resistant shigellosis as well as the development of resistance in other enteric bacteria through reductions in bystander exposure. As *Shigella* vaccines are evaluated in large Phase III trials, data on antibiotic treatment should be carefully collected such that the impact of the vaccine on antibiotic use can be measured [1,29]. To quantify this impact, it will be important for vaccine effectiveness to be estimated against less-severe disease endpoints that account for the bulk of antibiotic use.

Our estimates provide realistic expectations for the reductions in diarrhea outcomes at the population level that could be achieved by *Shigella* vaccines under real-world introduction scenarios. Uniquely, we demonstrate that *Shigella* vaccines could provide important reductions in antibiotic use for severe and nonsevere *Shigella* diarrheal episodes, and exposures to bystander pathogens due to *Shigella* treatment, which improves the value proposition for a *Shigella* vaccine. However, unless antibiotic use practices for diarrhea change more broadly as clinical suspicion of *Shigella* decreases, a *Shigella* vaccine in isolation is unlikely to make an appreciable impact on overall antibiotic use or exposures for bystander pathogens. Coadministration or combination of a *Shigella* vaccine with other vaccines for diarrheal disease may be more effective at limiting antimicrobial resistance.

## Supporting information

S1 Protocol Statistical analysis plan. (PDF)

S1 Appendix Supplemental results. (PDF)

## Abbreviations

AMR: antimicrobial resistance
Cq: quantification cycle
EPI: Expanded Programme on Immunization
F/M: fluoroquinolone and macrolide
LMIC: low- and middle- income country
MAL-ED: Etiology, Risk Factors, and Interactions of Enteric Infections and Malnutrition and the Consequences for Child Health and Development
OR: odds ratio
PPC: preferred product characteristic
qPCR: quantitative polymerase chain reaction
TAC: TaqMan Array Card
WHO: World Health Organization
