# Supplementary material for: Burden of diarrhea and antibiotic use among children in low-resource settings preventable by Shigella vaccination: A simulation study
Source: PLoS Med. 2023 Nov 22;20(11):e1004271. doi: 10.1371/journal.pmed.1004271 (PMC10707565; doi:10.1371/journal.pmed.1004271)
Supplement: S1 Protocol — (PDF) [file pmed.1004271.s001.pdf]

Quantifying the impact of enteric vaccines in preventing diarrheal illnesses in children under age 2 in MAL-ED

Funding Source:
Wellcome Trust

Investigators:
Elizabeth T. Rogawski McQuade, PhD, MSPH; Assistant Professor; Emory University
James A. Platts-Mills, MD; Associate Professor; University of Virginia
Joseph A. Lewnard, PhD; Assistant Professor; University of California, Berkeley
Stephanie A. Brennhofer, MPH, MS, RDN; Senior Data Analyst; University of Virginia

Summary:
This analysis is the third step (Aim 2) in a larger project to estimate the impact of enteric vaccines on etiology-specific diarrhea, antibiotic use, and exposure of subclinical infections to antibiotics among children in low-resource settings. Specifically, this analysis will estimate the impact of introducing two-dose series enteric vaccines with varying vaccine schedules (6&9, 9&12, 12&15 months), levels of indirect protection, effects on severity but not incidence, and vaccine efficacies. We will begin the analyses by focusing on hypothetical *Shigella* vaccines, with plans to analyze vaccines against additional enteric pathogens (e.g. *Campylobacter*, norovirus, enterotoxigenic *Escherichia coli* (ETEC), and rotavirus (vaccine available)). As in the previous analyses, we will analyze existing data from 1,715 children from the Malnutrition and Enteric Disease (MAL-ED) study.

Based on the different scenarios outlined above, will extend the results from Aim 1a (attributing diarrheal episodes that cause treatment) and from Aim 1b (attributing bystander antibiotic exposures for subclinical pathogens to diarrheal pathogens). Analyses will be performed at the level of diarrheal episode (N=6,677) from Aim 1a. We will then merge in the number of subclinical pathogen exposures to antibiotics per diarrheal episode from Aim 1b. We will use the attributable fractions per episode (AFe) for diarrheal pathogens that were calculated in Aim 1a where a pathogen with an AFe >0.5 is considered the cause of the diarrheal illness. We will then simulate how much antibiotic exposure calculated in Aims 1a (https://osf.io/e4bqr/) and 1b (https://osf.io/3asxh/) can be prevented with a vaccine. To simulate, we will perform Monte Carlo simulations with random sampling of the dataset with replacement to a sample size of 50,000. 95% confidence intervals for all parameters will be estimated by bootstrap with 1,000 resamples.

Analyses:

<u>Probabilistically sample observed episodes as "prevented" by vaccine and re-calculate incidence</u>

We will start with a vaccine for *Shigella* with dosing at 6 and 9 months of age. We will assume 60% efficacy 14-days after administration of the second dose. For simplicity, we will assume 0% efficacy prior to this time point. The Monte Carlo simulation will then be performed over the dataset with 50,000 samples under two scenarios: vaccine and no vaccine. For the vaccine scenario, we will probabilistically assign whether diarrheal episodes attributable to *Shigella* (defined by AFe > 0.5) would have been prevented assuming 60% efficacy. It is important to note that *Shigella* infections increase with age therefore, 60% efficacy will not directly translate to a 60% reduction in diarrheal illnesses. Additionally, there is low natural immunity (~20%) against subsequent *Shigella* diarrhea when considering all serotypes and species, so prior infection will not be factored in (Rogawski McQuade 2020 J Infect Dis),

at least initially. In a later iteration of the analyses, we may incorporate potential clustering of prevented episodes within children (i.e. all episodes among 60% of children are prevented, rather than 60% of episodes prevented overall, regardless of the child). We may also incorporate the potential for herd immunity among children too young to be vaccinated, partial efficacy after a single vaccine dose, different vaccine schedules (6&9, 9&12, 12&15 months), effects on severity in addition to or instead of incidence, and varying levels of vaccine efficacy. For the no vaccine scenario, no *Shigella*-attributable episodes would be prevented. We will conduct a sensitivity analysis where we define shigella diarrhea by any detection of shigella rather than AFe > 0.5.

We will estimate the expected reductions due to a *Shigella* vaccine in the following metrics:

1. Antibiotic treated severe *Shigella* diarrhea episodes

$$\left(\frac{N_{antibiotic\ treated\ severe\ diarrhea\ episodes\ attributed\ to\ Shigella} * \frac{total\ number\ of\ diarrhea\ associated\ courses}{Total\ number\ of\ linked\ diarrhea\ associated\ courses}}{total\ child\ years\ of\ follow\ up}\right) * 100$$

2. Antibiotic treated *Shigella* diarrhea episodes

$$\left(\frac{N_{antibiotic\ treated\ diarrhea\ episodes\ attributed\ to\ Shigella} * \frac{total\ number\ of\ diarrhea\ associated\ courses}{Total\ number\ of\ linked\ diarrhea\ associated\ courses}}{total\ child\ years\ of\ follow\ up}\right) * 100$$

3. Antibiotic treated severe diarrhea episodes of any etiology

$$\left(\frac{N_{reported\ severe\ antibiotic\ treated\ diarrhea\ episodes * \frac{total\ number\ of\ diarrhea\ associated\ courses}{Total\ number\ of\ linked\ diarrhea\ associated\ courses}}}{total\ child\ years\ of\ follow\ up}\right) * 100$$

4. Antibiotic treated diarrhea episodes of any etiology

$$\left(\frac{N_{reported\ antibiotic\ treated\ diarrhea\ episodes * \frac{total\ number\ of\ diarrhea\ associated\ courses}{Total\ number\ of\ linked\ diarrhea\ associated\ courses}}}{total\ child\ years\ of\ follow\ up}\right) * 100$$

5. Antibiotic courses overall

$$\left(\frac{N_{reported\ antibiotic\ courses} * \frac{total\ number\ of\ diarrhea\ associated\ courses}{Total\ number\ of\ linked\ diarrhea\ associated\ courses}}{total\ child\ years\ of\ follow\ up}\right) * 100$$

6. Antibiotic exposures to subclinical pathogens due to treatment of *Shigella*

$$\left(\frac{N\ times\ any\ bacterial\ pathogen\ exposed\ to\ abx\ during\ diarrhea\ caused\ by\ Shigella * \frac{total\ number\ of\ diarrhea\ associated\ courses}{Total\ number\ of\ linked\ diarrhea\ associated\ courses}}{Total\ number\ of\ linked\ courses}\right) * \left(\frac{Total\ number\ of\ courses}{Total\ child\ years\ of\ follow\ up}\right) * 100$$

7. Antibiotic exposures to subclinical pathogens overall

$$\left(\frac{N\ times\ any\ bacterial\ pathogen\ exposed\ to\ antibiotic * \frac{total\ number\ of\ diarrhea\ associated\ courses}{Total\ number\ of\ linked\ diarrhea\ associated\ courses}}{Total\ number\ of\ linked\ courses}\right) * \left(\frac{Total\ number\ of\ courses}{Total\ child\ years\ of\ follow\ up}\right) * 100$$

To estimate the expected reductions in #1-7 listed above, we will compare the relative and absolute differences between the vaccine and no vaccine simulations.

Relative difference, is expressed as a ratio of the metric between the vaccine simulations and the no vaccine simulation, which will be calculated as:

$$\frac{vaccine\ simulation}{no\ vaccine\ simulation}$$

Or the percent change as:

$$\frac{vaccine\ simulation - no\ vaccine\ simulation}{no\ vaccine\ simulation}$$

Absolute difference, which captures the true distance between two values, will be calculated as:

$$|\ vaccine\ simulation - no\ vaccine\ simulation\ |$$

As a secondary outcome, we will estimate the average age of *Shigella* diarrhea under the vaccine and no vaccine scenarios to determine whether the vaccine would be expected to increase the average age of disease.

Future directions:

Once we have the *Shigella* vaccine simulation running, we will compare the impact of a *Shigella* vaccine to that for other enteric vaccines, most importantly rotavirus. We may also include a comparison to a broad stewardship intervention that is not targeted to a specific pathogen.

Added Sept 12, 2022:

We will estimate the expected reductions in diarrheal episodes caused by *Shigella* and any etiology (regardless of whether the episode was treated):

1. Severe *Shigella* diarrhea episodes

$$\left(\frac{N_{severe\ diarrhea\ episodes\ attributed\ to\ Shigella} * \frac{total\ number\ of\ diarrhea\ episodes}{Total\ number\ of\ diarrhea\ episodes\ validly\ tested\ by\ TAC}}{total\ child\ years\ of\ follow\ up}\right) * 100$$

2. *Shigella* diarrhea episodes

$$\left(\frac{N_{diarrhea\ episodes\ attributed\ to\ Shigella} * \frac{total\ number\ of\ diarrhea\ episodes}{Total\ number\ of\ diarrhea\ episodes\ validly\ tested\ by\ TAC}}{total\ child\ years\ of\ follow\ up}\right) * 100$$

3. Severe diarrhea episodes of any etiology

$$\left(\frac{N_{reported\ severe\ diarrhea\ episodes} * \frac{total\ number\ of\ diarrhea\ episodes}{Total\ number\ of\ diarrhea\ episodes\ validly\ tested\ by\ TAC}}{total\ child\ years\ of\ follow\ up}\right) * 100$$

4. Diarrhea episodes of any etiology

$$\left(\frac{N_{reported\ diarrhea\ episodes} * \frac{total\ number\ of\ diarrhea\ episodes}{Total\ number\ of\ diarrhea\ episodes\ validly\ tested\ by\ TAC}}{total\ child\ years\ of\ follow\ up}\right) * 100$$
