# Supplementary material for: Burden of diarrhea and antibiotic use among children in low-resource settings preventable by Shigella vaccination: A simulation study
Source: PLoS Med. 2023 Nov 22;20(11):e1004271. doi: 10.1371/journal.pmed.1004271 (PMC10707565; doi:10.1371/journal.pmed.1004271)
Supplement: S1 Appendix — (PDF) [file pmed.1004271.s002.pdf]

S1 Appendix

Burden of diarrhea and antibiotic use among children in low-resource settings preventable by *Shigella* vaccination: a simulation study

Stephanie A Brennhofer; James A Platts-Mills; Joseph A Lewnard; Jie Liu; Eric R Houpt; Elizabeth T Rogawski McQuade

Supplemental results

Table A. Relative percent reductions in diarrhea outcomes for 5 *Shigella* vaccine scenarios with 60% and 80% full vaccine efficacies against severe *Shigella* diarrhea and no indirect or boosting protection. 4

Table B. Absolute (incidence rate differences) and relative (incidence rate ratios) differences in diarrhea outcomes for 5 *Shigella* vaccine scenarios compared to the no vaccine scenario with added indirect protection (60% and 80% full vaccine efficacies against severe *Shigella* diarrhea). 5

Table C. Absolute (incidence rate differences) and relative (incidence rate ratios) differences in diarrhea outcomes for 5 *Shigella* vaccine scenarios compared to the no vaccine scenario with added boosting protection (60% and 80% full vaccine efficacies against severe *Shigella* diarrhea). 6

Table D. Absolute (incidence rate differences) and relative (incidence rate ratios) differences in diarrhea outcomes for 5 *Shigella* vaccine scenarios compared to the no vaccine scenario with added indirect and boosting protection (60% and 80% full vaccine efficacies against severe *Shigella* diarrhea). 7

Table E. Relative percent reductions in diarrhea outcomes among the 9- and 12-month *Shigella* vaccine dosing scenario with 60% full vaccine efficacy against severe *Shigella* diarrhea. 8

Table F. Relative percent reductions in fluroquinolone and macrolide (F/M) use outcomes for 5 *Shigella* vaccine scenarios with 60% and 80% full vaccine efficacies against severe *Shigella* diarrhea and no indirect or boosting protection. 9

Table G. Absolute (incidence rate differences) and relative (incidence rate ratios) differences in fluoroquinolone and macrolide (F/M) outcomes for 5 *Shigella* vaccine scenarios compared to the no vaccine scenario with added indirect protection (60% and 80% full vaccine efficacies against severe *Shigella* diarrhea). 10

Table H. Absolute (incidence rate differences) and relative (incidence rate ratios) differences in fluoroquinolone and macrolide (F/M) outcomes for 5 *Shigella* vaccine scenarios compared to the no vaccine scenario with added boosting protection (60% and 80% full vaccine efficacies against severe *Shigella* diarrhea). 11

Table I. Absolute (incidence rate differences) and relative (incidence rate ratios) differences in fluoroquinolone and macrolide (F/M) outcomes for 5 *Shigella* vaccine scenarios compared to the no vaccine scenario with added indirect and boosting protection (60% and 80% full vaccine efficacies against severe *Shigella* diarrhea)... 12

Table J. Relative percent reductions in fluroquinolone and macrolide (F/M) use outcomes among the 9- and 12-month *Shigella* vaccine dosing scenario with 60% full vaccine efficacy against severe *Shigella* diarrhea. 13

Table K. Absolute (incidence rate differences) and relative (incidence rate ratios) differences in antibiotic use outcomes (any drug class) for 5 *Shigella* vaccine scenarios compared to the no vaccine scenario with 60% and 80% full vaccine efficacies against severe *Shigella* diarrhea and no indirect and boosting protection. 14

Fig A. Relative percent reductions in antibiotic use outcomes (any drug class) for 5 *Shigella* vaccine scenarios with 60% and 80% full vaccine efficacies against severe *Shigella* diarrhea and no indirect or boosting protection... 16

Table L. Relative percent reductions in antibiotic use outcomes (any drug class) for 5 *Shigella* vaccine scenarios with 60% and 80% full vaccine efficacies against severe *Shigella* diarrhea and no indirect or boosting protection... 17

Table M. Absolute (incidence rate differences) and relative (incidence rate ratios) differences in antibiotic use outcomes (any drug class) for 5 *Shigella* vaccine scenarios compared to the no vaccine scenario with added indirect protection (60% and 80% full vaccine efficacies against severe *Shigella* diarrhea). 18

Table N. Absolute (incidence rate differences) and relative (incidence rate ratios) differences in antibiotic use outcomes (any drug class) for 5 *Shigella* vaccine scenarios compared to the no vaccine scenario with added boosting protection (60% and 80% full vaccine efficacies against severe *Shigella* diarrhea).......... 19

Table O. Absolute (incidence rate differences) and relative (incidence rate ratios) differences in antibiotic use outcomes (any drug class) for 5 *Shigella* vaccine scenarios compared to the no vaccine scenario with added indirect and boosting protection (60% and 80% full vaccine efficacies against severe *Shigella* diarrhea). 20

Fig B. Relative percent reductions in antibiotic use outcomes (any drug class) among the 9- and 12-month *Shigella* vaccine dosing scenario with 60% full vaccine efficacy against severe *Shigella* diarrhea. 21

Table P. Relative percent reductions in antibiotic use outcomes (any drug class) among the 9- and 12-month *Shigella* vaccine dosing scenario with 60% full vaccine efficacy against severe *Shigella* diarrhea. 22

Table Q. Absolute (incidence rate differences) and relative (incidence rate ratios) differences in diarrhea outcomes among the 9- and 12-month *Shigella* vaccine dosing scenario compared to the no vaccine scenario with 60% full vaccine efficacy against severe *Shigella* diarrhea by site. 23

Fig C. Relative percent reductions in diarrhea outcomes among the 9- and 12-month *Shigella* vaccine dosing scenario with 60% full vaccine efficacy against severe *Shigella* diarrhea by site.......... 24

Table R. Absolute (incidence rate differences) and relative (incidence rate ratios) differences in fluoroquinolone/macrolide (F/M) outcomes among the 9- and 12-month *Shigella* vaccine dosing scenario compared to the no vaccine scenario with 60% full vaccine efficacy against severe *Shigella* diarrhea by site. 25

Fig D. Relative percent reductions in fluoroquinolone and macrolide (F/M) use among the 9- and 12-month *Shigella* vaccine dosing scenario with 60% full vaccine efficacy against severe *Shigella* diarrhea by site. 27

Table S. Absolute (incidence rate differences) and relative (incidence rate ratios) differences in antibiotic use outcomes (any drug class) among the 9- and 12-month *Shigella* vaccine dosing scenario compared to the no vaccine scenario with 60% full vaccine efficacy against severe *Shigella* diarrhea by site. 28

Table A. Relative percent reductions in diarrhea outcomes for 5 *Shigella* vaccine scenarios with 60% and 80% full vaccine efficacies against severe *Shigella* diarrhea and no indirect or boosting protection.

Vaccine scenario and efficacy outcome	Relative percent reduction	
	60% VE (95% CI)	80% VE (95% CI)
One dose - 6 months		
Severe *Shigella* diarrhea episodes	-53.1 (-57.1, -48.3)	-71.0 (-75.9, -64.8)
Severe diarrhea episodes of any etiology	-4.2 (-5.1, -3.3)	-5.6 (-6.9, -4.4)
Shigella diarrhea episodes	-40.8 (-41.7, -39.9)	-60.2 (-61.3, -59.2)
Diarrhea episodes of any etiology	-4.6 (-4.9, -4.2)	-6.7 (-7.3, -6.2)
One dose - 9 months		
Severe *Shigella* diarrhea episodes	-41.0 (-46.8, -34.3)	-54.7 (-62.9, -46.4)
Severe diarrhea episodes of any etiology	-3.2 (-4.1, -2.4)	-4.3 (-5.5, -3.2)
Shigella diarrhea episodes	-37.4 (-38.5, -36.1)	-55.3 (-56.8, -53.6)
Diarrhea episodes of any etiology	-4.2 (-4.5, -3.8)	-6.2 (-6.7, -5.6)
Two doses - 6 months & 9 months		
Severe *Shigella* diarrhea episodes	-44.3 (-49.5, -38.7)	-59.4 (-66.0, -52.3)
Severe diarrhea episodes of any etiology	-3.5 (-4.3, -2.7)	-4.6 (-5.7, -3.6)
Shigella diarrhea episodes	-38.7 (-39.7, -37.8)	-57.3 (-58.5, -56.0)
Diarrhea episodes of any etiology	-4.3 (-4.7, -4.0)	-6.4 (-7.0, -5.9)
Two doses - 9 months & 12 months		
Severe *Shigella* diarrhea episodes	-34.0 (-40.5, -27.9)	-46.0 (-54.1, -38.1)
Severe diarrhea episodes of any etiology	-2.7 (-3.5, -2.0)	-3.6 (-4.7, -2.7)
Shigella diarrhea episodes	-34.5 (-35.8, -33.1)	-51.0 (-52.8, -49.1)
Diarrhea episodes of any etiology	-3.8 (-4.2, -3.5)	-5.7 (-6.2, -5.2)
Two doses - 12 months & 15 months		
Severe *Shigella* diarrhea episodes	-22.7 (-28.6, -17.2)	-31.0 (-38.6, -23.4)
Severe diarrhea episodes of any etiology	-1.8 (-2.4, -1.2)	-2.4 (-3.2, -1.7)
Shigella diarrhea episodes	-28.0 (-29.5, -26.5)	-41.6 (-43.7, -39.4)
Diarrhea episodes of any etiology	-3.1 (-3.4, -2.8)	-4.6 (-5.1, -4.2)

Data are also reported in Fig 1. VE= vaccine efficacy; CI = confidence interval

Table B. Absolute (incidence rate differences) and relative (incidence rate ratios) differences in diarrhea outcomes for 5 *Shigella* vaccine scenarios compared to the no vaccine scenario with added indirect protection (60% and 80% full vaccine efficacies against severe *Shigella* diarrhea).

Vaccine scenario and efficacy outcome	Incidence rate difference (cases per 100 child-years)		Incidence rate ratio	
	60% VE (95% CI)	80% VE (95% CI)	60% VE (95% CI)	80% VE (95% CI)
One dose - 6 months				
Severe *Shigella* diarrhea episodes	-2.0 (-2.4, -1.5)	-2.6 (-3.2, -2.0)	0.46 (0.42, 0.50)	0.28 (0.23, 0.33)
Severe diarrhea episodes of any etiology	-2.0 (-2.4, -1.5)	-2.6 (-3.2, -2.0)	0.96 (0.95, 0.97)	0.94 (0.93, 0.95)
Shigella diarrhea episodes	-12.7 (-13.7, -11.5)	-18.6 (-20.2, -17.0)	0.59 (0.58, 0.59)	0.39 (0.38, 0.40)
Diarrhea episodes of any etiology	-12.7 (-13.7, -11.5)	-18.6 (-20.2, -17.0)	0.95 (0.95, 0.96)	0.93 (0.93, 0.94)
One dose - 9 months				
Severe *Shigella* diarrhea episodes	-1.6 (-2.0, -1.2)	-2.1 (-2.6, -1.6)	0.56 (0.50, 0.61)	0.42 (0.34, 0.49)
Severe diarrhea episodes of any etiology	-1.6 (-2.0, -1.2)	-2.1 (-2.6, -1.6)	0.97 (0.96, 0.97)	0.95 (0.94, 0.96)
Shigella diarrhea episodes	-12.0 (-13.0, -10.9)	-17.5 (-19.0, -15.9)	0.61 (0.60, 0.62)	0.43 (0.42, 0.44)
Diarrhea episodes of any etiology	-12.0 (-13.0, -10.9)	-17.5 (-19.0, -15.9)	0.96 (0.95, 0.96)	0.94 (0.93, 0.94)
Two doses - 6 months & 9 months				
Severe *Shigella* diarrhea episodes	-1.6 (-2.1, -1.3)	-2.2 (-2.7, -1.7)	0.55 (0.50, 0.60)	0.40 (0.33, 0.47)
Severe diarrhea episodes of any etiology	-1.6 (-2.1, -1.3)	-2.2 (-2.7, -1.7)	0.96 (0.96, 0.97)	0.95 (0.94, 0.96)
Shigella diarrhea episodes	-12.0 (-13.1, -10.9)	-17.7 (-19.2, -16.1)	0.61 (0.60, 0.62)	0.42 (0.41, 0.43)
Diarrhea episodes of any etiology	-12.0 (-13.1, -10.9)	-17.7 (-19.2, -16.1)	0.96 (0.95, 0.96)	0.94 (0.93, 0.94)
Two doses - 9 months & 12 months				
Severe *Shigella* diarrhea episodes	-1.4 (-1.7, -1.0)	-1.8 (-2.3, -1.3)	0.62 (0.57, 0.68)	0.51 (0.43, 0.58)
Severe diarrhea episodes of any etiology	-1.4 (-1.7, -1.0)	-1.8 (-2.3, -1.3)	0.97 (0.96, 0.98)	0.96 (0.95, 0.97)
Shigella diarrhea episodes	-11.1 (-12.1, -10.0)	-16.2 (-17.7, -14.6)	0.64 (0.63, 0.65)	0.47 (0.46, 0.49)
Diarrhea episodes of any etiology	-11.1 (-12.1, -10.0)	-16.2 (-17.7, -14.6)	0.96 (0.96, 0.96)	0.94 (0.94, 0.95)
Two doses - 12 months & 15 months				
Severe *Shigella* diarrhea episodes	-1.0 (-1.3, -0.7)	-1.3 (-1.7, -1.0)	0.72 (0.67, 0.77)	0.64 (0.57, 0.70)
Severe diarrhea episodes of any etiology	-1.0 (-1.3, -0.7)	-1.3 (-1.7, -1.0)	0.98 (0.97, 0.98)	0.97 (0.96, 0.98)
Shigella diarrhea episodes	-9.8 (-10.7, -8.9)	-14.0 (-15.3, -12.6)	0.68 (0.67, 0.69)	0.54 (0.53, 0.56)
Diarrhea episodes of any etiology	-9.8 (-10.7, -8.9)	-14.0 (-15.3, -12.6)	0.96 (0.96, 0.97)	0.95 (0.94, 0.95)

VE= vaccine efficacy; CI = confidence interval

Table C. Absolute (incidence rate differences) and relative (incidence rate ratios) differences in diarrhea outcomes for 5 *Shigella* vaccine scenarios compared to the no vaccine scenario with added boosting protection (60% and 80% full vaccine efficacies against severe *Shigella* diarrhea).

Vaccine scenario and efficacy outcome	Incidence rate difference (cases per 100 child-years)		Incidence rate ratio	
	60% VE (95% CI)	80% VE (95% CI)	60% VE (95% CI)	80% VE (95% CI)
One dose - 6 months				
Severe *Shigella* diarrhea episodes	-1.9 (-2.4, -1.5)	-2.6 (-3.2, -2.0)	0.47 (0.43, 0.51)	0.29 (0.24, 0.35)
Severe diarrhea episodes of any etiology	-1.9 (-2.4, -1.5)	-2.6 (-3.2, -2.0)	0.96 (0.95, 0.97)	0.94 (0.93, 0.96)
Shigella diarrhea episodes	-13.0 (-14.2, -11.9)	-19.0 (-20.7, -17.3)	0.58 (0.57, 0.59)	0.38 (0.37, 0.39)
Diarrhea episodes of any etiology	-13.0 (-14.2, -11.9)	-19.0 (-20.7, -17.3)	0.95 (0.95, 0.96)	0.93 (0.93, 0.94)
One dose - 9 months				
Severe *Shigella* diarrhea episodes	-1.5 (-1.9, -1.1)	-2.0 (-2.5, -1.5)	0.59 (0.53, 0.65)	0.45 (0.37, 0.53)
Severe diarrhea episodes of any etiology	-1.5 (-1.9, -1.1)	-2.0 (-2.5, -1.5)	0.97 (0.96, 0.98)	0.96 (0.95, 0.97)
Shigella diarrhea episodes	-12.4 (-13.6, -11.3)	-18.0 (-19.6, -16.3)	0.59 (0.58, 0.61)	0.41 (0.40, 0.43)
Diarrhea episodes of any etiology	-12.4 (-13.6, -11.3)	-18.0 (-19.6, -16.3)	0.95 (0.95, 0.96)	0.93 (0.93, 0.94)
Two doses - 6 months & 9 months				
Severe *Shigella* diarrhea episodes	-1.6 (-2.0, -1.2)	-2.2 (-2.7, -1.6)	0.55 (0.50, 0.61)	0.40 (0.34, 0.47)
Severe diarrhea episodes of any etiology	-1.6 (-2.0, -1.2)	-2.2 (-2.7, -1.6)	0.97 (0.96, 0.97)	0.95 (0.94, 0.96)
Shigella diarrhea episodes	-12.9 (-14.0, -11.7)	-18.6 (-20.2, -16.9)	0.58 (0.57, 0.59)	0.39 (0.38, 0.41)
Diarrhea episodes of any etiology	-12.9 (-14.0, -11.7)	-18.6 (-20.2, -16.9)	0.95 (0.95, 0.96)	0.93 (0.93, 0.94)
Two doses - 9 months & 12 months				
Severe *Shigella* diarrhea episodes	-1.2 (-1.6, -0.9)	-1.7 (-2.1, -1.3)	0.66 (0.59, 0.72)	0.54 (0.45, 0.61)
Severe diarrhea episodes of any etiology	-1.2 (-1.6, -0.9)	-1.7 (-2.1, -1.3)	0.97 (0.97, 0.98)	0.96 (0.95, 0.97)
Shigella diarrhea episodes	-12.2 (-13.4, -11.0)	-17.3 (-19.0, -15.7)	0.60 (0.59, 0.62)	0.43 (0.42, 0.45)
Diarrhea episodes of any etiology	-12.2 (-13.4, -11.0)	-17.3 (-19.0, -15.7)	0.96 (0.95, 0.96)	0.94 (0.93, 0.94)
Two doses - 12 months & 15 months				
Severe *Shigella* diarrhea episodes	-0.8 (-1.1, -0.6)	-1.1 (-1.5, -0.8)	0.77 (0.71, 0.82)	0.68 (0.61, 0.76)
Severe diarrhea episodes of any etiology	-0.8 (-1.1, -0.6)	-1.1 (-1.5, -0.8)	0.98 (0.98, 0.99)	0.98 (0.97, 0.98)
Shigella diarrhea episodes	-10.6 (-11.6, -9.4)	-14.7 (-16.2, -13.1)	0.66 (0.64, 0.67)	0.52 (0.50, 0.54)
Diarrhea episodes of any etiology	-10.6 (-11.6, -9.4)	-14.7 (-16.2, -13.1)	0.96 (0.96, 0.97)	0.95 (0.94, 0.95)

VE= vaccine efficacy; CI = confidence interval

Table D. Absolute (incidence rate differences) and relative (incidence rate ratios) differences in diarrhea outcomes for 5 *Shigella* vaccine scenarios compared to the no vaccine scenario with added indirect and boosting protection (60% and 80% full vaccine efficacies against severe *Shigella* diarrhea).

Vaccine scenario and efficacy outcome	Incidence rate difference (cases per 100 child-years)		Incidence rate ratio	
	60% VE (95% CI)	80% VE (95% CI)	60% VE (95% CI)	80% VE (95% CI)
One dose - 6 months				
Severe *Shigella* diarrhea episodes	-2.0 (-2.4, -1.5)	-2.6 (-3.2, -2.0)	0.46 (0.42, 0.50)	0.28 (0.23, 0.33)
Severe diarrhea episodes of any etiology	-2.0 (-2.4, -1.5)	-2.6 (-3.2, -2.0)	0.96 (0.95, 0.97)	0.94 (0.93, 0.95)
Shigella diarrhea episodes	-13.2 (-14.4, -12.0)	-19.1 (-20.8, -17.4)	0.57 (0.56, 0.58)	0.38 (0.37, 0.39)
Diarrhea episodes of any etiology	-13.2 (-14.4, -12.0)	-19.1 (-20.8, -17.4)	0.95 (0.95, 0.96)	0.93 (0.92, 0.94)
One dose - 9 months				
Severe *Shigella* diarrhea episodes	-1.6 (-2.0, -1.2)	-2.1 (-2.6, -1.6)	0.55 (0.50, 0.61)	0.41 (0.34, 0.49)
Severe diarrhea episodes of any etiology	-1.6 (-2.0, -1.2)	-2.1 (-2.6, -1.6)	0.97 (0.96, 0.97)	0.95 (0.94, 0.96)
Shigella diarrhea episodes	-13.0 (-14.2, -11.8)	-18.5 (-20.1, -16.9)	0.58 (0.57, 0.59)	0.40 (0.38, 0.41)
Diarrhea episodes of any etiology	-13.0 (-14.2, -11.8)	-18.5 (-20.1, -16.9)	0.95 (0.95, 0.96)	0.93 (0.93, 0.94)
Two doses - 6 months & 9 months				
Severe *Shigella* diarrhea episodes	-1.6 (-2.1, -1.3)	-2.2 (-2.7, -1.7)	0.54 (0.50, 0.59)	0.39 (0.33, 0.45)
Severe diarrhea episodes of any etiology	-1.6 (-2.1, -1.3)	-2.2 (-2.7, -1.7)	0.96 (0.96, 0.97)	0.95 (0.94, 0.96)
Shigella diarrhea episodes	-13.0 (-14.2, -11.9)	-18.7 (-20.4, -17.1)	0.57 (0.56, 0.58)	0.39 (0.38, 0.40)
Diarrhea episodes of any etiology	-13.0 (-14.2, -11.9)	-18.7 (-20.4, -17.1)	0.95 (0.95, 0.96)	0.93 (0.93, 0.94)
Two doses - 9 months & 12 months				
Severe *Shigella* diarrhea episodes	-1.4 (-1.7, -1.0)	-1.8 (-2.3, -1.4)	0.62 (0.56, 0.68)	0.50 (0.43, 0.58)
Severe diarrhea episodes of any etiology	-1.4 (-1.7, -1.0)	-1.8 (-2.3, -1.4)	0.97 (0.96, 0.98)	0.96 (0.95, 0.97)
Shigella diarrhea episodes	-12.8 (-14.0, -11.6)	-17.9 (-19.5, -16.2)	0.58 (0.57, 0.60)	0.42 (0.40, 0.43)
Diarrhea episodes of any etiology	-12.8 (-14.0, -11.6)	-17.9 (-19.5, -16.2)	0.95 (0.95, 0.96)	0.93 (0.93, 0.94)
Two doses - 12 months & 15 months				
Severe *Shigella* diarrhea episodes	-1.0 (-1.3, -0.8)	-1.3 (-1.7, -1.0)	0.71 (0.66, 0.76)	0.63 (0.56, 0.70)
Severe diarrhea episodes of any etiology	-1.0 (-1.3, -0.8)	-1.3 (-1.7, -1.0)	0.98 (0.97, 0.98)	0.97 (0.96, 0.98)
Shigella diarrhea episodes	-11.8 (-13.0, -10.7)	-16.0 (-17.5, -14.4)	0.61 (0.60, 0.63)	0.48 (0.46, 0.50)
Diarrhea episodes of any etiology	-11.8 (-13.0, -10.7)	-16.0 (-17.5, -14.4)	0.96 (0.95, 0.96)	0.94 (0.94, 0.95)

VE= vaccine efficacy; CI = confidence interval

Table E. Relative percent reductions in diarrhea outcomes among the 9- and 12-month *Shigella* vaccine dosing scenario with 60% full vaccine efficacy against severe *Shigella* diarrhea.

Vaccine scenario and efficacy outcome	Relative percent reduction
Direct	
Severe *Shigella* diarrhea episodes	-34.0 (-40.5, -27.9)
Severe diarrhea episodes of any etiology	-2.7 (-3.5, -2.0)
Shigella diarrhea episodes	-34.5 (-35.8, -33.1)
Diarrhea episodes of any etiology	-3.8 (-4.2, -3.5)
Direct + Indirect	
Severe *Shigella* diarrhea episodes	-37.5 (-43.1, -31.8)
Severe diarrhea episodes of any etiology	-2.9 (-3.7, -2.3)
Shigella diarrhea episodes	-36.2 (-37.2, -35.2)
Diarrhea episodes of any etiology	-4.0 (-4.4, -3.7)
Direct + Boost	
Severe *Shigella* diarrhea episodes	-34.4 (-40.8, -28.5)
Severe diarrhea episodes of any etiology	-2.7 (-3.5, -2.0)
Shigella diarrhea episodes	-39.9 (-41.3, -38.4)
Diarrhea episodes of any etiology	-4.5 (-4.9, -4.1)
Direct + Indirect + Boost	
Severe *Shigella* diarrhea episodes	-37.9 (-43.5, -32.2)
Severe diarrhea episodes of any etiology	-3.0 (-3.8, -2.3)
Shigella diarrhea episodes	-41.7 (-42.9, -40.3)
Diarrhea episodes of any etiology	-4.7 (-5.1, -4.3)

Data are also reported in Fig 2. VE= vaccine efficacy; CI = confidence interval

Table F. Relative percent reductions in fluroquinolone and macrolide (F/M) use outcomes for 5 *Shigella* vaccine scenarios with 60% and 80% full vaccine efficacies against severe *Shigella* diarrhea and no indirect or boosting protection.

Vaccine scenario and efficacy outcome	Relative percent reduction	
	60% VE (95% CI)	80% VE (95% CI)
One dose - 6 months		
F/M courses for severe *Shigella* diarrhea episodes	-50.8 (-59.2, -38.7)	-67.8 (-78.1, -52.9)
F/M courses for severe diarrhea episodes of any etiology	-7.1 (-10.7, -4.1)	-9.4 (-14.2, -5.5)
F/M courses for *Shigella* diarrhea episodes	-41.1 (-42.7, -39.3)	-60.7 (-62.3, -58.6)
F/M courses for diarrhea episodes of any etiology	-9.0 (-10.2, -8.0)	-13.3 (-15.0, -11.7)
F/M courses overall	-3.2 (-3.6, -2.7)	-4.7 (-5.3, -4.1)
F/M exposures to bystander pathogens due to *Shigella* treatment	-40.9 (-42.8, -38.7)	-60.3 (-62.3, -57.8)
F/M exposures to bystander pathogens overall	-3.6 (-4.1, -3.1)	-5.3 (-6.1, -4.5)
One dose - 9 months		
F/M courses for severe *Shigella* diarrhea episodes	-46.7 (-56.2, -33.4)	-62.5 (-74.7, -45.1)
F/M courses for severe diarrhea episodes of any etiology	-6.6 (-10.0, -3.5)	-8.8 (-13.4, -4.8)
F/M courses for *Shigella* diarrhea episodes	-38.9 (-40.9, -36.8)	-57.4 (-59.8, -54.8)
F/M courses for diarrhea episodes of any etiology	-8.5 (-9.6, -7.5)	-12.6 (-14.2, -11.1)
F/M courses overall	-3.0 (-3.4, -2.6)	-4.4 (-5.0, -3.8)
F/M exposures to bystander pathogens due to *Shigella* treatment	-38.3 (-40.5, -36.0)	-56.6 (-59.4, -53.6)
F/M exposures to bystander pathogens overall	-3.3 (-3.9, -2.8)	-5.0 (-5.7, -4.2)
Two doses - 6 months & 9 months		
F/M courses for severe *Shigella* diarrhea episodes	-48.7 (-57.1, -36.4)	-65.0 (-76.4, -49.0)
F/M courses for severe diarrhea episodes of any etiology	-6.7 (-10.4, -3.9)	-9.0 (-13.7, -5.2)
F/M courses for *Shigella* diarrhea episodes	-40.0 (-41.6, -38.2)	-59.0 (-61.0, -56.9)
F/M courses for diarrhea episodes of any etiology	-8.8 (-9.9, -7.7)	-13.0 (-14.6, -11.4)
F/M courses overall	-3.1 (-3.5, -2.7)	-4.5 (-5.2, -3.9)
F/M exposures to bystander pathogens due to *Shigella* treatment	-39.6 (-41.4, -37.5)	-58.5 (-60.7, -56.1)
F/M exposures to bystander pathogens overall	-3.5 (-4.0, -3.0)	-5.1 (-5.9, -4.4)
Two doses - 9 months & 12 months		
F/M courses for severe *Shigella* diarrhea episodes	-35.4 (-48.9, -22.9)	-47.8 (-64.7, -31.5)
F/M courses for severe diarrhea episodes of any etiology	-5.0 (-7.6, -2.7)	-6.7 (-10.2, -3.8)
F/M courses for *Shigella* diarrhea episodes	-35.8 (-38, -33.5)	-53.1 (-56.2, -49.8)
F/M courses for diarrhea episodes of any etiology	-7.8 (-8.9, -6.8)	-11.6 (-13.2, -10.2)
F/M courses overall	-2.8 (-3.1, -2.4)	-4.1 (-4.7, -3.5)
F/M exposures to bystander pathogens due to *Shigella* treatment	-34.9 (-37.3, -32.3)	-51.7 (-55.3, -48.2)
F/M exposures to bystander pathogens overall	-3.1 (-3.6, -2.6)	-4.5 (-5.3, -3.8)
Two doses - 12 months & 15 months		
F/M courses for severe *Shigella* diarrhea episodes	-25.8 (-38.2, -15.1)	-34.7 (-51.3, -20.8)
F/M courses for severe diarrhea episodes of any etiology	-3.6 (-5.9, -1.8)	-4.9 (-7.9, -2.4)
F/M courses for *Shigella* diarrhea episodes	-29.8 (-32.3, -27.2)	-44.3 (-47.9, -40.5)
F/M courses for diarrhea episodes of any etiology	-6.5 (-7.5, -5.7)	-9.7 (-11.2, -8.4)
F/M courses overall	-2.3 (-2.7, -1.9)	-3.4 (-3.9, -2.9)
F/M exposures to bystander pathogens due to *Shigella* treatment	-28.2 (-31.2, -25.2)	-42.0 (-46.1, -37.9)
F/M exposures to bystander pathogens overall	-2.5 (-2.9, -2.0)	-3.7 (-4.3, -3.1)

Data are also reported in Fig 3. VE= vaccine efficacy; CI = confidence interval; F/M = fluoroquinolones/macrolides

Table G. Absolute (incidence rate differences) and relative (incidence rate ratios) differences in fluoroquinolone and macrolide (F/M) outcomes for 5 *Shigella* vaccine scenarios compared to the no vaccine scenario with added indirect protection (60% and 80% full vaccine efficacies against severe *Shigella* diarrhea).

Vaccine scenario and efficacy outcome	Incidence rate difference (cases per 100 child-years)		Incidence rate ratio	
	60% VE (95% CI)	80% VE (95% CI)	60% VE (95% CI)	80% VE (95% CI)
One dose - 6 months				
F/M courses for *Shigella* diarrhea episodes	-3.4 (-4.0, -2.9)	-5.0 (-5.9, -4.2)	0.59 (0.57, 0.60)	0.39 (0.37, 0.41)
F/M courses for diarrhea episodes of any etiology	-3.4 (-4.0, -2.9)	-5.0 (-5.9, -4.2)	0.91 (0.90, 0.92)	0.87 (0.85, 0.88)
F/M courses overall	-3.4 (-4.0, -2.9)	-5.0 (-5.9, -4.2)	0.97 (0.96, 0.97)	0.95 (0.95, 0.96)
One dose - 9 months				
F/M courses for *Shigella* diarrhea episodes	-3.3 (-3.9, -2.8)	-4.9 (-5.7, -4.1)	0.60 (0.58, 0.61)	0.41 (0.39, 0.43)
F/M courses for diarrhea episodes of any etiology	-3.3 (-3.9, -2.8)	-4.9 (-5.7, -4.1)	0.91 (0.90, 0.92)	0.87 (0.86, 0.89)
F/M courses overall	-3.3 (-3.9, -2.8)	-4.9 (-5.7, -4.1)	0.97 (0.96, 0.97)	0.95 (0.95, 0.96)
Two doses - 6 months & 9 months				
F/M courses for *Shigella* diarrhea episodes	-3.3 (-3.9, -2.8)	-4.9 (-5.8, -4.1)	0.60 (0.58, 0.61)	0.41 (0.39, 0.43)
F/M courses for diarrhea episodes of any etiology	-3.3 (-3.9, -2.8)	-4.9 (-5.8, -4.1)	0.91 (0.90, 0.92)	0.87 (0.85, 0.89)
F/M courses overall	-3.3 (-3.9, -2.8)	-4.9 (-5.8, -4.1)	0.97 (0.96, 0.97)	0.95 (0.95, 0.96)
Two doses - 9 months & 12 months				
F/M courses for *Shigella* diarrhea episodes	-3.1 (-3.6, -2.6)	-4.5 (-5.3, -3.8)	0.63 (0.61, 0.65)	0.46 (0.43, 0.48)
F/M courses for diarrhea episodes of any etiology	-3.1 (-3.6, -2.6)	-4.5 (-5.3, -3.8)	0.92 (0.91, 0.93)	0.88 (0.87, 0.90)
F/M courses overall	-3.1 (-3.6, -2.6)	-4.5 (-5.3, -3.8)	0.97 (0.97, 0.98)	0.96 (0.95, 0.96)
Two doses - 12 months & 15 months				
F/M courses for *Shigella* diarrhea episodes	-2.7 (-3.2, -2.3)	-4.0 (-4.6, -3.3)	0.67 (0.65, 0.69)	0.52 (0.50, 0.56)
F/M courses for diarrhea episodes of any etiology	-2.7 (-3.2, -2.3)	-4.0 (-4.6, -3.3)	0.93 (0.92, 0.94)	0.90 (0.88, 0.91)
F/M courses overall	-2.7 (-3.2, -2.3)	-4.0 (-4.6, -3.3)	0.97 (0.97, 0.98)	0.96 (0.96, 0.97)

VE= vaccine efficacy; CI = confidence interval; F/M = fluoroquinolones/macrolides

Table H. Absolute (incidence rate differences) and relative (incidence rate ratios) differences in fluoroquinolone and macrolide (F/M) outcomes for 5 *Shigella* vaccine scenarios compared to the no vaccine scenario with added boosting protection (60% and 80% full vaccine efficacies against severe *Shigella* diarrhea).

Vaccine scenario and efficacy outcome	Incidence rate difference (cases per 100 child-years)		Incidence rate ratio	
	60% VE (95% CI)	80% VE (95% CI)	60% VE (95% CI)	80% VE (95% CI)
One dose - 6 months				
F/M courses for *Shigella* diarrhea episodes	-3.6 (-4.2, -3.0)	-5.2 (-6.0, -4.3)	0.57 (0.56, 0.59)	0.38 (0.36, 0.40)
F/M courses for diarrhea episodes of any etiology	-3.6 (-4.2, -3.0)	-5.2 (-6.0, -4.3)	0.91 (0.89, 0.92)	0.86 (0.85, 0.88)
F/M courses overall	-3.6 (-4.2, -3.0)	-5.2 (-6.0, -4.3)	0.97 (0.96, 0.97)	0.95 (0.95, 0.96)
One dose - 9 months				
F/M courses for *Shigella* diarrhea episodes	-3.5 (-4.2, -2.9)	-5.1 (-6.0, -4.2)	0.57 (0.55, 0.59)	0.39 (0.36, 0.42)
F/M courses for diarrhea episodes of any etiology	-3.5 (-4.2, -2.9)	-5.1 (-6.0, -4.2)	0.91 (0.89, 0.92)	0.87 (0.85, 0.88)
F/M courses overall	-3.5 (-4.2, -2.9)	-5.1 (-6.0, -4.2)	0.97 (0.96, 0.97)	0.95 (0.95, 0.96)
Two doses - 6 months & 9 months				
F/M courses for *Shigella* diarrhea episodes	-3.6 (-4.3, -3.1)	-5.2 (-6.1, -4.4)	0.56 (0.55, 0.58)	0.37 (0.35, 0.39)
F/M courses for diarrhea episodes of any etiology	-3.6 (-4.3, -3.1)	-5.2 (-6.1, -4.4)	0.90 (0.89, 0.92)	0.86 (0.84, 0.88)
F/M courses overall	-3.6 (-4.3, -3.1)	-5.2 (-6.1, -4.4)	0.97 (0.96, 0.97)	0.95 (0.94, 0.96)
Two doses - 9 months & 12 months				
F/M courses for *Shigella* diarrhea episodes	-3.5 (-4.1, -2.9)	-4.9 (-5.8, -4.1)	0.58 (0.55, 0.61)	0.41 (0.37, 0.45)
F/M courses for diarrhea episodes of any etiology	-3.5 (-4.1, -2.9)	-4.9 (-5.8, -4.1)	0.91 (0.90, 0.92)	0.87 (0.85, 0.89)
F/M courses overall	-3.5 (-4.1, -2.9)	-4.9 (-5.8, -4.1)	0.97 (0.96, 0.97)	0.95 (0.95, 0.96)
Two doses - 12 months & 15 months				
F/M courses for *Shigella* diarrhea episodes	-3.1 (-3.7, -2.5)	-4.3 (-5.1, -3.5)	0.63 (0.60, 0.66)	0.49 (0.44, 0.53)
F/M courses for diarrhea episodes of any etiology	-3.1 (-3.7, -2.5)	-4.3 (-5.1, -3.5)	0.92 (0.91, 0.93)	0.89 (0.87, 0.90)
F/M courses overall	-3.1 (-3.7, -2.5)	-4.3 (-5.1, -3.5)	0.97 (0.97, 0.98)	0.96 (0.95, 0.97)

VE= vaccine efficacy; CI = confidence interval; F/M = fluoroquinolones/macrolides

Table I. Absolute (incidence rate differences) and relative (incidence rate ratios) differences in fluoroquinolone and macrolide (F/M) outcomes for 5 *Shigella* vaccine scenarios compared to the no vaccine scenario with added indirect and boosting protection (60% and 80% full vaccine efficacies against severe *Shigella* diarrhea).

Vaccine scenario and efficacy outcome	Incidence rate difference (cases per 100 child-years)		Incidence rate ratio	
	60% VE (95% CI)	80% VE (95% CI)	60% VE (95% CI)	80% VE (95% CI)
One dose - 6 months				
F/M courses for *Shigella* diarrhea episodes	-3.6 (-4.2, -3.0)	-5.2 (-6.1, -4.3)	0.57 (0.55, 0.59)	0.37 (0.36, 0.39)
F/M courses for diarrhea episodes of any etiology	-3.6 (-4.2, -3.0)	-5.2 (-6.1, -4.3)	0.91 (0.89, 0.92)	0.86 (0.85, 0.88)
F/M courses overall	-3.6 (-4.2, -3.0)	-5.2 (-6.1, -4.3)	0.97 (0.96, 0.97)	0.95 (0.94, 0.96)
One dose - 9 months				
F/M courses for *Shigella* diarrhea episodes	-3.6 (-4.3, -3.1)	-5.2 (-6.1, -4.3)	0.56 (0.54, 0.58)	0.38 (0.35, 0.40)
F/M courses for diarrhea episodes of any etiology	-3.6 (-4.3, -3.1)	-5.2 (-6.1, -4.3)	0.90 (0.89, 0.92)	0.86 (0.85, 0.88)
F/M courses overall	-3.6 (-4.3, -3.1)	-5.2 (-6.1, -4.3)	0.97 (0.96, 0.97)	0.95 (0.95, 0.96)
Two doses - 6 months & 9 months				
F/M courses for *Shigella* diarrhea episodes	-3.7 (-4.3, -3.1)	-5.2 (-6.1, -4.4)	0.56 (0.54, 0.58)	0.37 (0.35, 0.39)
F/M courses for diarrhea episodes of any etiology	-3.7 (-4.3, -3.1)	-5.2 (-6.1, -4.4)	0.90 (0.89, 0.92)	0.86 (0.84, 0.88)
F/M courses overall	-3.7 (-4.3, -3.1)	-5.2 (-6.1, -4.4)	0.97 (0.96, 0.97)	0.95 (0.94, 0.96)
Two doses - 9 months & 12 months				
F/M courses for *Shigella* diarrhea episodes	-3.6 (-4.2, -3.0)	-5.0 (-5.9, -4.2)	0.57 (0.54, 0.59)	0.40 (0.36, 0.43)
F/M courses for diarrhea episodes of any etiology	-3.6 (-4.2, -3.0)	-5.0 (-5.9, -4.2)	0.91 (0.89, 0.92)	0.87 (0.85, 0.88)
F/M courses overall	-3.6 (-4.2, -3.0)	-5.0 (-5.9, -4.2)	0.97 (0.96, 0.97)	0.95 (0.95, 0.96)
Two doses - 12 months & 15 months				
F/M courses for *Shigella* diarrhea episodes	-3.3 (-4.0, -2.8)	-4.5 (-5.4, -3.8)	0.60 (0.57, 0.63)	0.45 (0.42, 0.49)
F/M courses for diarrhea episodes of any etiology	-3.3 (-4.0, -2.8)	-4.5 (-5.4, -3.8)	0.91 (0.90, 0.92)	0.88 (0.86, 0.90)
F/M courses overall	-3.3 (-4.0, -2.8)	-4.5 (-5.4, -3.8)	0.97 (0.96, 0.97)	0.96 (0.95, 0.96)

VE= vaccine efficacy; CI = confidence interval; F/M = fluoroquinolones/macrolides

Table J. Relative percent reductions in fluroquinolone and macrolide (F/M) use outcomes among the 9- and 12-month *Shigella* vaccine dosing scenario with 60% full vaccine efficacy against severe *Shigella* diarrhea.

Vaccine scenario and efficacy outcome	Relative percent reduction
Direct	
F/M courses for severe *Shigella* diarrhea episodes	-35.4 (-48.9, -22.9)
F/M courses for severe diarrhea episodes of any etiology	-5.0 (-7.6, -2.7)
F/M courses for *Shigella* diarrhea episodes	-35.8 (-38.0, -33.5)
F/M courses for diarrhea episodes of any etiology	-7.8 (-8.9, -6.8)
F/M courses overall	-2.8 (-3.1, -2.4)
F/M exposures to bystander pathogens due to *Shigella* treatment	-34.9 (-37.3, -32.3)
F/M exposures to bystander pathogens overall	-3.1 (-3.6, -2.6)
Direct + Indirect	
F/M courses for severe *Shigella* diarrhea episodes	-38.9 (-51.3, -27.0)
F/M courses for severe diarrhea episodes of any etiology	-5.5 (-8.1, -3.2)
F/M courses for *Shigella* diarrhea episodes	-37.1 (-39.1, -35.1)
F/M courses for diarrhea episodes of any etiology	-8.1 (-9.2, -7.1)
F/M courses overall	-2.9 (-3.3, -2.5)
F/M exposures to bystander pathogens due to *Shigella* treatment	-36.5 (-38.5, -34.3)
F/M exposures to bystander pathogens overall	-3.2 (-3.7, -2.7)
Direct + Boost	
F/M courses for severe *Shigella* diarrhea episodes	-35.6 (-48.9, -23.3)
F/M courses for severe diarrhea episodes of any etiology	-5.0 (-7.7, -2.8)
F/M courses for *Shigella* diarrhea episodes	-41.8 (-44.6, -39.0)
F/M courses for diarrhea episodes of any etiology	-9.2 (-10.4, -7.9)
F/M courses overall	-3.2 (-3.7, -2.8)
F/M exposures to bystander pathogens due to *Shigella* treatment	-41.1 (-44.4, -38.4)
F/M exposures to bystander pathogens overall	-3.6 (-4.2, -3.0)
Direct + Indirect + Boost	
F/M courses for severe *Shigella* diarrhea episodes	-39.2 (-51.0, -27.9)
F/M courses for severe diarrhea episodes of any etiology	-5.5 (-8.2, -3.4)
F/M courses for *Shigella* diarrhea episodes	-43.2 (-45.7, -40.6)
F/M courses for diarrhea episodes of any etiology	-9.5 (-10.7, -8.3)
F/M courses overall	-3.3 (-3.8, -2.9)
F/M exposures to bystander pathogens due to *Shigella* treatment	-42.9 (-45.8, -40.1)
F/M exposures to bystander pathogens overall	-3.7 (-4.3, -3.2)

Data are also reported in Fig 4. VE= vaccine efficacy; CI = confidence interval; F/M = fluoroquinolones/macrolides

Table K. Absolute (incidence rate differences) and relative (incidence rate ratios) differences in antibiotic use outcomes (any drug class) for 5 *Shigella* vaccine scenarios compared to the no vaccine scenario with 60% and 80% full vaccine efficacies against severe *Shigella* diarrhea and no indirect and boosting protection.

Vaccine scenario and efficacy outcome	Incidence rate difference (cases per 100 child-years)		Incidence rate ratio	
	60% VE (95% CI)	80% VE (95% CI)	60% VE (95% CI)	80% VE (95% CI)
One dose - 6 months				
Antibiotic courses for severe *Shigella* diarrhea episodes	-1.4 (-1.8, -1.0)	-1.9 (-2.4, -1.3)	0.47 (0.42, 0.53)	0.29 (0.23, 0.37)
Antibiotic courses for severe diarrhea episodes of any etiology	-1.4 (-1.8, -1.0)	-1.9 (-2.4, -1.3)	0.95 (0.94, 0.96)	0.94 (0.92, 0.95)
Antibiotic courses for *Shigella* diarrhea episodes	-6.8 (-7.6, -6.1)	-10.0 (-11.1, -8.9)	0.58 (0.57, 0.60)	0.39 (0.38, 0.41)
Antibiotic courses for diarrhea episodes of any etiology	-6.8 (-7.6, -6.1)	-10.0 (-11.1, -8.9)	0.94 (0.93, 0.94)	0.91 (0.90, 0.92)
Antibiotic courses overall	-6.8 (-7.6, -6.1)	-10.0 (-11.1, -8.9)	0.99 (0.99, 0.99)	0.98 (0.98, 0.99)
Antibiotic exposures to bystander pathogens due to *Shigella* treatment	-13.2 (-15.0, -11.6)	-19.3 (-22.1, -17.0)	0.59 (0.57, 0.61)	0.40 (0.38, 0.42)
Antibiotic exposures to bystander pathogens overall	-13.2 (-15.0, -11.6)	-19.3 (-22.1, -17.0)	0.99 (0.98, 0.99)	0.98 (0.98, 0.98)
One dose - 9 months				
Antibiotic courses for severe *Shigella* diarrhea episodes	-1.1 (-1.5, -0.8)	-1.5 (-2.0, -1.0)	0.57 (0.50, 0.66)	0.43 (0.33, 0.54)
Antibiotic courses for severe diarrhea episodes of any etiology	-1.1 (-1.5, -0.8)	-1.5 (-2.0, -1.0)	0.96 (0.95, 0.97)	0.95 (0.93, 0.96)
Antibiotic courses for *Shigella* diarrhea episodes	-6.2 (-7.0, -5.5)	-9.2 (-10.2, -8.2)	0.62 (0.60, 0.64)	0.44 (0.42, 0.47)
Antibiotic courses for diarrhea episodes of any etiology	-6.2 (-7.0, -5.5)	-9.2 (-10.2, -8.2)	0.94 (0.94, 0.95)	0.92 (0.91, 0.93)
Antibiotic courses overall	-6.2 (-7.0, -5.5)	-9.2 (-10.2, -8.2)	0.99 (0.99, 0.99)	0.98 (0.98, 0.99)
Antibiotic exposures to bystander pathogens due to *Shigella* treatment	-12.1 (-13.9, -10.6)	-17.9 (-20.3, -15.6)	0.62 (0.60, 0.64)	0.44 (0.42, 0.47)
Antibiotic exposures to bystander pathogens overall	-12.1 (-13.9, -10.6)	-17.9 (-20.3, -15.6)	0.99 (0.99, 0.99)	0.98 (0.98, 0.98)
Two doses - 6 months & 9 months				
Antibiotic courses for severe *Shigella* diarrhea episodes	-1.2 (-1.6, -0.9)	-1.7 (-2.1, -1.2)	0.53 (0.47, 0.59)	0.37 (0.30, 0.46)
Antibiotic courses for severe diarrhea episodes of any etiology	-1.2 (-1.6, -0.9)	-1.7 (-2.1, -1.2)	0.96 (0.94, 0.97)	0.94 (0.93, 0.96)
Antibiotic courses for *Shigella* diarrhea episodes	-6.5 (-7.3, -5.8)	-9.5 (-10.6, -8.5)	0.60 (0.59, 0.62)	0.42 (0.40, 0.44)
Antibiotic courses for diarrhea episodes of any etiology	-6.5 (-7.3, -5.8)	-9.5 (-10.6, -8.5)	0.94 (0.93, 0.95)	0.91 (0.90, 0.92)
Antibiotic courses overall	-6.5 (-7.3, -5.8)	-9.5 (-10.6, -8.5)	0.99 (0.99, 0.99)	0.98 (0.98, 0.99)
Antibiotic exposures to bystander pathogens due to *Shigella* treatment	-12.6 (-14.4, -11.0)	-18.6 (-21.2, -16.3)	0.61 (0.59, 0.62)	0.42 (0.40, 0.44)
Antibiotic exposures to bystander pathogens overall	-12.6 (-14.4, -11.0)	-18.6 (-21.2, -16.3)	0.99 (0.98, 0.99)	0.98 (0.98, 0.98)
Two doses - 9 months & 12 months				
Antibiotic courses for severe *Shigella* diarrhea episodes	-0.9 (-1.2, -0.6)	-1.2 (-1.6, -0.8)	0.67 (0.58, 0.74)	0.55 (0.45, 0.65)
Antibiotic courses for severe diarrhea episodes of any etiology	-0.9 (-1.2, -0.6)	-1.2 (-1.6, -0.8)	0.97 (0.96, 0.98)	0.96 (0.95, 0.97)
Antibiotic courses for *Shigella* diarrhea episodes	-5.6 (-6.3, -5.0)	-8.3 (-9.3, -7.4)	0.66 (0.64, 0.68)	0.49 (0.47, 0.52)
Antibiotic courses for diarrhea episodes of any etiology	-5.6 (-6.3, -5.0)	-8.3 (-9.3, -7.4)	0.95 (0.94, 0.95)	0.92 (0.92, 0.93)
Antibiotic courses overall	-5.6 (-6.3, -5.0)	-8.3 (-9.3, -7.4)	0.99 (0.99, 0.99)	0.99 (0.98, 0.99)
Antibiotic exposures to bystander pathogens due to *Shigella* treatment	-10.9 (-12.5, -9.5)	-16.1 (-18.4, -14.1)	0.66 (0.64, 0.68)	0.50 (0.47, 0.53)
Antibiotic exposures to bystander pathogens overall	-10.9 (-12.5, -9.5)	-16.1 (-18.4, -14.1)	0.99 (0.99, 0.99)	0.98 (0.98, 0.99)

Two doses - 12 months & 15 months				
Antibiotic courses for severe *Shigella* diarrhea episodes	-0.6 (-0.8, -0.3)	-0.8 (-1.1, -0.5)	0.78 (0.71, 0.85)	0.70 (0.61, 0.79)
Antibiotic courses for severe diarrhea episodes of any etiology	-0.6 (-0.8, -0.3)	-0.8 (-1.1, -0.5)	0.98 (0.97, 0.99)	0.97 (0.96, 0.98)
Antibiotic courses for *Shigella* diarrhea episodes	-4.5 (-5.1, -4.0)	-6.7 (-7.6, -5.9)	0.72 (0.70, 0.75)	0.59 (0.56, 0.62)
Antibiotic courses for diarrhea episodes of any etiology	-4.5 (-5.1, -4.0)	-6.7 (-7.6, -5.9)	0.96 (0.95, 0.96)	0.94 (0.93, 0.95)
Antibiotic courses overall	-4.5 (-5.1, -4.0)	-6.7 (-7.6, -5.9)	0.99 (0.99, 0.99)	0.99 (0.99, 0.99)
Antibiotic exposures to bystander pathogens due to *Shigella* treatment	-8.5 (-9.9, -7.3)	-12.7 (-14.7, -10.9)	0.73 (0.71, 0.76)	0.60 (0.57, 0.64)
Antibiotic exposures to bystander pathogens overall	-8.5 (-9.9, -7.3)	-12.7 (-14.7, -10.9)	0.99 (0.99, 0.99)	0.99 (0.98, 0.99)

VE= vaccine efficacy; CI = confidence interval

Fig A. Relative percent reductions in antibiotic use outcomes (any drug class) for 5 *Shigella* vaccine scenarios with 60% and 80% full vaccine efficacies against severe *Shigella* diarrhea and no indirect or boosting protection.

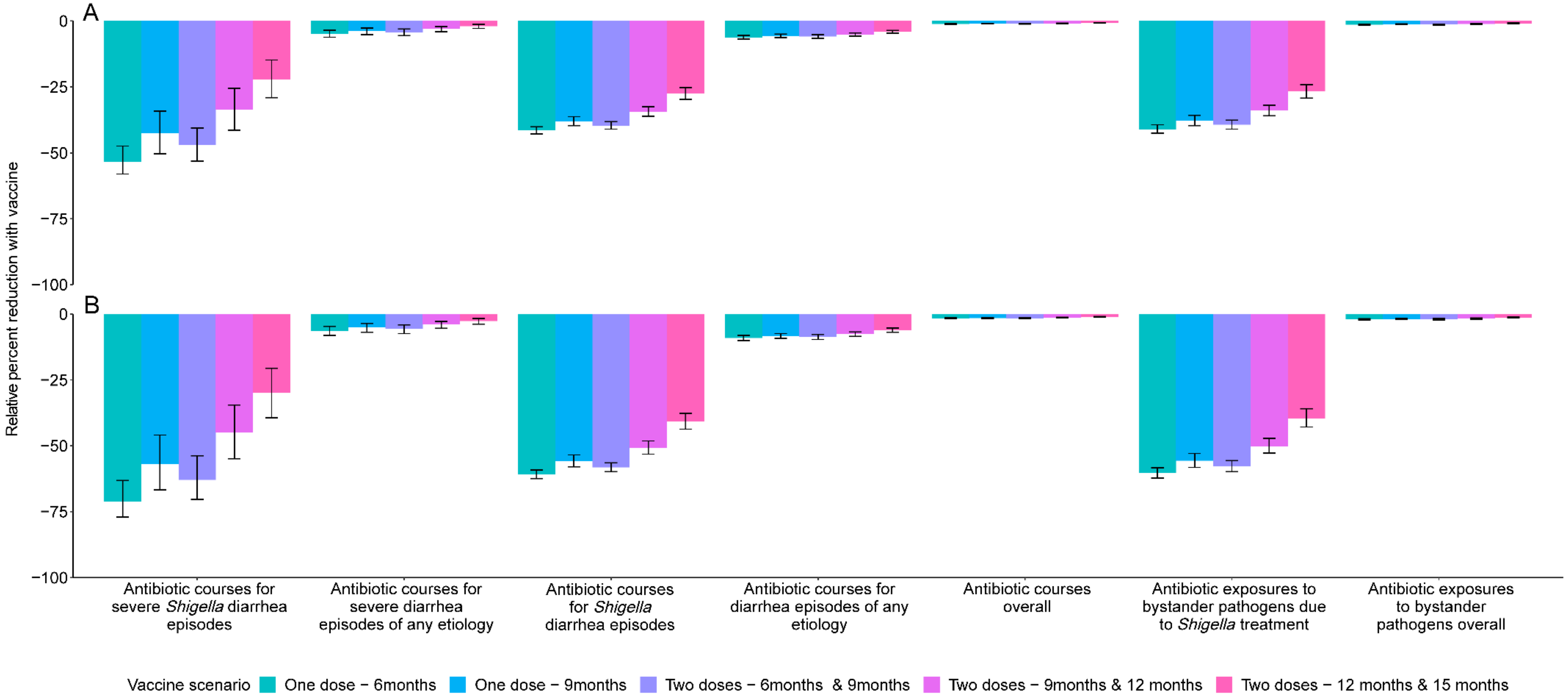

Caption: The black lines represent 95% confidence intervals.

Table L. Relative percent reductions in antibiotic use outcomes (any drug class) for 5 *Shigella* vaccine scenarios with 60% and 80% full vaccine efficacies against severe *Shigella* diarrhea and no indirect or boosting protection.

Vaccine scenario and efficacy outcome	Relative percent reduction	
	60% VE (95% CI)	80% VE (95% CI)
One dose - 6 months		
Antibiotic courses for severe *Shigella* diarrhea episodes	-53.3 (-58.0, -47.4)	-71.1 (-77.1, -63.2)
Antibiotic courses for severe diarrhea episodes of any etiology	-4.8 (-6.2, -3.5)	-6.4 (-8.2, -4.7)
Antibiotic courses for *Shigella* diarrhea episodes	-41.5 (-42.9, -40.1)	-60.9 (-62.5, -59.2)
Antibiotic courses for diarrhea episodes of any etiology	-6.2 (-6.9, -5.5)	-9.1 (-10.1, -8.1)
Antibiotic courses overall	-1.1 (-1.3, -1.0)	-1.7 (-1.8, -1.5)
Antibiotic exposures to bystander pathogens due to *Shigella* treatment	-41.0 (-42.6, -39.4)	-60.3 (-62.2, -58.3)
Antibiotic exposures to bystander pathogens overall	-1.4 (-1.6, -1.2)	-2.0 (-2.3, -1.8)
One dose - 9 months		
Antibiotic courses for severe *Shigella* diarrhea episodes	-42.5 (-50.4, -34.2)	-57.0 (-66.7, -45.9)
Antibiotic courses for severe diarrhea episodes of any etiology	-3.8 (-5.2, -2.6)	-5.1 (-6.9, -3.6)
Antibiotic courses for *Shigella* diarrhea episodes	-38.0 (-39.7, -36.3)	-55.9 (-58.0, -53.5)
Antibiotic courses for diarrhea episodes of any etiology	-5.7 (-6.3, -5.0)	-8.3 (-9.3, -7.4)
Antibiotic courses overall	-1.0 (-1.2, -0.9)	-1.5 (-1.7, -1.4)
Antibiotic exposures to bystander pathogens due to *Shigella* treatment	-37.8 (-39.7, -35.8)	-55.7 (-58.2, -52.9)
Antibiotic exposures to bystander pathogens overall	-1.3 (-1.4, -1.1)	-1.9 (-2.1, -1.6)
Two doses - 6 months & 9 months		
Antibiotic courses for severe *Shigella* diarrhea episodes	-47.0 (-53.2, -40.7)	-63.0 (-70.4, -53.8)
Antibiotic courses for severe diarrhea episodes of any etiology	-4.2 (-5.6, -3.1)	-5.7 (-7.4, -4.1)
Antibiotic courses for *Shigella* diarrhea episodes	-39.6 (-41.0, -38.2)	-58.2 (-59.8, -56.5)
Antibiotic courses for diarrhea episodes of any etiology	-5.9 (-6.6, -5.3)	-8.7 (-9.6, -7.7)
Antibiotic courses overall	-1.1 (-1.2, -1.0)	-1.6 (-1.8, -1.4)
Antibiotic exposures to bystander pathogens due to *Shigella* treatment	-39.3 (-41.0, -37.6)	-57.9 (-59.9, -55.6)
Antibiotic exposures to bystander pathogens overall	-1.3 (-1.5, -1.2)	-2.0 (-2.2, -1.7)
Two doses - 9 months & 12 months		
Antibiotic courses for severe *Shigella* diarrhea episodes	-33.5 (-41.5, -25.6)	-44.9 (-54.9, -34.6)
Antibiotic courses for severe diarrhea episodes of any etiology	-3.0 (-4.1, -2.1)	-4.0 (-5.4, -2.8)
Antibiotic courses for *Shigella* diarrhea episodes	-34.5 (-36.2, -32.4)	-50.9 (-53.2, -48.2)
Antibiotic courses for diarrhea episodes of any etiology	-5.1 (-5.7, -4.6)	-7.6 (-8.5, -6.8)
Antibiotic courses overall	-0.9 (-1.0, -0.8)	-1.4 (-1.5, -1.2)
Antibiotic exposures to bystander pathogens due to *Shigella* treatment	-33.9 (-35.9, -31.9)	-50.2 (-52.8, -47.1)
Antibiotic exposures to bystander pathogens overall	-1.1 (-1.3, -1.0)	-1.7 (-1.9, -1.5)
Two doses - 12 months & 15 months		
Antibiotic courses for severe *Shigella* diarrhea episodes	-22.1 (-29.1, -14.8)	-29.9 (-39.4, -20.6)
Antibiotic courses for severe diarrhea episodes of any etiology	-2.0 (-2.8, -1.2)	-2.7 (-3.8, -1.7)
Antibiotic courses for *Shigella* diarrhea episodes	-27.5 (-29.8, -25.3)	-40.7 (-43.7, -37.7)
Antibiotic courses for diarrhea episodes of any etiology	-4.1 (-4.6, -3.6)	-6.1 (-6.9, -5.4)
Antibiotic courses overall	-0.7 (-0.8, -0.7)	-1.1 (-1.3, -1.0)
Antibiotic exposures to bystander pathogens due to *Shigella* treatment	-26.6 (-29.2, -24.1)	-39.6 (-42.9, -35.9)
Antibiotic exposures to bystander pathogens overall	-0.9 (-1.0, -0.8)	-1.3 (-1.5, -1.2)

Data are also reported in Fig A. VE= vaccine efficacy; CI = confidence interval

Table M. Absolute (incidence rate differences) and relative (incidence rate ratios) differences in antibiotic use outcomes (any drug class) for 5 *Shigella* vaccine scenarios compared to the no vaccine scenario with added indirect protection (60% and 80% full vaccine efficacies against severe *Shigella* diarrhea).

Vaccine scenario and efficacy outcome	Incidence rate difference (cases per 100 child-years)		Incidence rate ratio	
	60% VE (95% CI)	80% VE (95% CI)	60% VE (95% CI)	80% VE (95% CI)
One dose - 6 months				
Antibiotic courses for *Shigella* diarrhea episodes	-6.9 (-7.7, -6.2)	-10.1 (-11.3, -9.0)	0.58 (0.57, 0.59)	0.39 (0.37, 0.40)
Antibiotic courses for diarrhea episodes of any etiology	-6.9 (-7.7, -6.2)	-10.1 (-11.3, -9.0)	0.94 (0.93, 0.94)	0.91 (0.90, 0.92)
Antibiotic courses overall	-6.9 (-7.7, -6.2)	-10.1 (-11.3, -9.0)	0.99 (0.99, 0.99)	0.98 (0.98, 0.99)
One dose - 9 months				
Antibiotic courses for *Shigella* diarrhea episodes	-6.5 (-7.3, -5.9)	-9.5 (-10.6, -8.5)	0.60 (0.59, 0.61)	0.42 (0.41, 0.44)
Antibiotic courses for diarrhea episodes of any etiology	-6.5 (-7.3, -5.9)	-9.5 (-10.6, -8.5)	0.94 (0.93, 0.95)	0.91 (0.90, 0.92)
Antibiotic courses overall	-6.5 (-7.3, -5.9)	-9.5 (-10.6, -8.5)	0.99 (0.99, 0.99)	0.98 (0.98, 0.99)
Two doses - 6 months & 9 months				
Antibiotic courses for *Shigella* diarrhea episodes	-6.6 (-7.4, -5.9)	-9.6 (-10.7, -8.6)	0.60 (0.59, 0.61)	0.41 (0.40, 0.43)
Antibiotic courses for diarrhea episodes of any etiology	-6.6 (-7.4, -5.9)	-9.6 (-10.7, -8.6)	0.94 (0.93, 0.95)	0.91 (0.90, 0.92)
Antibiotic courses overall	-6.6 (-7.4, -5.9)	-9.6 (-10.7, -8.6)	0.99 (0.99, 0.99)	0.98 (0.98, 0.99)
Two doses - 9 months & 12 months				
Antibiotic courses for *Shigella* diarrhea episodes	-6.0 (-6.6, -5.3)	-8.7 (-9.6, -7.7)	0.64 (0.62, 0.65)	0.47 (0.45, 0.50)
Antibiotic courses for diarrhea episodes of any etiology	-6.0 (-6.6, -5.3)	-8.7 (-9.6, -7.7)	0.95 (0.94, 0.95)	0.92 (0.91, 0.93)
Antibiotic courses overall	-6.0 (-6.6, -5.3)	-8.7 (-9.6, -7.7)	0.99 (0.99, 0.99)	0.99 (0.98, 0.99)
Two doses - 12 months & 15 months				
Antibiotic courses for *Shigella* diarrhea episodes	-5.2 (-5.9, -4.7)	-7.4 (-8.3, -6.6)	0.68 (0.66, 0.70)	0.55 (0.52, 0.57)
Antibiotic courses for diarrhea episodes of any etiology	-5.2 (-5.9, -4.7)	-7.4 (-8.3, -6.6)	0.95 (0.95, 0.96)	0.93 (0.92, 0.94)
Antibiotic courses overall	-5.2 (-5.9, -4.7)	-7.4 (-8.3, -6.6)	0.99 (0.99, 0.99)	0.99 (0.99, 0.99)

VE= vaccine efficacy; CI = confidence interval

Table N. Absolute (incidence rate differences) and relative (incidence rate ratios) differences in antibiotic use outcomes (any drug class) for 5 *Shigella* vaccine scenarios compared to the no vaccine scenario with added boosting protection (60% and 80% full vaccine efficacies against severe *Shigella* diarrhea).

Vaccine scenario and efficacy outcome	Incidence rate difference (cases per 100 child-years)		Incidence rate ratio	
	60% VE (95% CI)	80% VE (95% CI)	60% VE (95% CI)	80% VE (95% CI)
One dose - 6 months				
Antibiotic courses for *Shigella* diarrhea episodes	-7.1 (-7.9, -6.3)	-10.2 (-11.5, -9.2)	0.57 (0.55, 0.58)	0.37 (0.36, 0.39)
Antibiotic courses for diarrhea episodes of any etiology	-7.1 (-7.9, -6.3)	-10.2 (-11.5, -9.2)	0.94 (0.93, 0.94)	0.91 (0.90, 0.92)
Antibiotic courses overall	-7.1 (-7.9, -6.3)	-10.2 (-11.5, -9.2)	0.99 (0.99, 0.99)	0.98 (0.98, 0.98)
One dose - 9 months				
Antibiotic courses for *Shigella* diarrhea episodes	-6.7 (-7.6, -6.0)	-9.6 (-10.8, -8.6)	0.59 (0.57, 0.61)	0.41 (0.39, 0.43)
Antibiotic courses for diarrhea episodes of any etiology	-6.7 (-7.6, -6.0)	-9.6 (-10.8, -8.6)	0.94 (0.93, 0.95)	0.91 (0.90, 0.92)
Antibiotic courses overall	-6.7 (-7.6, -6.0)	-9.6 (-10.8, -8.6)	0.99 (0.99, 0.99)	0.98 (0.98, 0.99)
Two doses - 6 months & 9 months				
Antibiotic courses for *Shigella* diarrhea episodes	-7.0 (-7.9, -6.2)	-10.1 (-11.2, -9.0)	0.57 (0.56, 0.59)	0.39 (0.37, 0.40)
Antibiotic courses for diarrhea episodes of any etiology	-7.0 (-7.9, -6.2)	-10.1 (-11.2, -9.0)	0.94 (0.93, 0.94)	0.91 (0.90, 0.92)
Antibiotic courses overall	-7.0 (-7.9, -6.2)	-10.1 (-11.2, -9.0)	0.99 (0.99, 0.99)	0.98 (0.98, 0.99)
Two doses - 9 months & 12 months				
Antibiotic courses for *Shigella* diarrhea episodes	-6.5 (-7.2, -5.7)	-9.2 (-10.2, -8.2)	0.60 (0.58, 0.63)	0.44 (0.41, 0.47)
Antibiotic courses for diarrhea episodes of any etiology	-6.5 (-7.2, -5.7)	-9.2 (-10.2, -8.2)	0.94 (0.93, 0.95)	0.92 (0.91, 0.93)
Antibiotic courses overall	-6.5 (-7.2, -5.7)	-9.2 (-10.2, -8.2)	0.99 (0.99, 0.99)	0.98 (0.98, 0.99)
Two doses - 12 months & 15 months				
Antibiotic courses for *Shigella* diarrhea episodes	-5.5 (-6.3, -4.8)	-7.7 (-8.7, -6.7)	0.67 (0.64, 0.69)	0.53 (0.50, 0.57)
Antibiotic courses for diarrhea episodes of any etiology	-5.5 (-6.3, -4.8)	-7.7 (-8.7, -6.7)	0.95 (0.94, 0.96)	0.93 (0.92, 0.94)
Antibiotic courses overall	-5.5 (-6.3, -4.8)	-7.7 (-8.7, -6.7)	0.99 (0.99, 0.99)	0.99 (0.99, 0.99)

VE= vaccine efficacy; CI = confidence interval

Table O. Absolute (incidence rate differences) and relative (incidence rate ratios) differences in antibiotic use outcomes (any drug class) for 5 *Shigella* vaccine scenarios compared to the no vaccine scenario with added indirect and boosting protection (60% and 80% full vaccine efficacies against severe *Shigella* diarrhea).

Vaccine scenario and efficacy outcome	Incidence rate difference (cases per 100 child-years)		Incidence rate ratio	
	60% VE (95% CI)	80% VE (95% CI)	60% VE (95% CI)	80% VE (95% CI)
One dose - 6 months				
Antibiotic courses for *Shigella* diarrhea episodes	-7.2 (-8.0, -6.4)	-10.3 (-11.5, -9.2)	0.56 (0.55, 0.57)	0.37 (0.35, 0.38)
Antibiotic courses for diarrhea episodes of any etiology	-7.2 (-8.0, -6.4)	-10.3 (-11.5, -9.2)	0.93 (0.93, 0.94)	0.91 (0.90, 0.92)
Antibiotic courses overall	-7.2 (-8.0, -6.4)	-10.3 (-11.5, -9.2)	0.99 (0.99, 0.99)	0.98 (0.98, 0.98)
One dose - 9 months				
Antibiotic courses for *Shigella* diarrhea episodes	-7.0 (-7.9, -6.3)	-10.0 (-11.1, -8.9)	0.57 (0.55, 0.58)	0.39 (0.37, 0.41)
Antibiotic courses for diarrhea episodes of any etiology	-7.0 (-7.9, -6.3)	-10.0 (-11.1, -8.9)	0.94 (0.93, 0.94)	0.91 (0.90, 0.92)
Antibiotic courses overall	-7.0 (-7.9, -6.3)	-10.0 (-11.1, -8.9)	0.99 (0.99, 0.99)	0.98 (0.98, 0.99)
Two doses - 6 months & 9 months				
Antibiotic courses for *Shigella* diarrhea episodes	-7.1 (-7.9, -6.3)	-10.1 (-11.3, -9.1)	0.57 (0.55, 0.58)	0.38 (0.36, 0.40)
Antibiotic courses for diarrhea episodes of any etiology	-7.1 (-7.9, -6.3)	-10.1 (-11.3, -9.1)	0.94 (0.93, 0.94)	0.91 (0.90, 0.92)
Antibiotic courses overall	-7.1 (-7.9, -6.3)	-10.1 (-11.3, -9.1)	0.99 (0.99, 0.99)	0.98 (0.98, 0.98)
Two doses - 9 months & 12 months				
Antibiotic courses for *Shigella* diarrhea episodes	-6.8 (-7.6, -6.1)	-9.5 (-10.6, -8.5)	0.58 (0.57, 0.61)	0.42 (0.40, 0.45)
Antibiotic courses for diarrhea episodes of any etiology	-6.8 (-7.6, -6.1)	-9.5 (-10.6, -8.5)	0.94 (0.93, 0.94)	0.91 (0.90, 0.92)
Antibiotic courses overall	-6.8 (-7.6, -6.1)	-9.5 (-10.6, -8.5)	0.99 (0.99, 0.99)	0.98 (0.98, 0.99)
Two doses - 12 months & 15 months				
Antibiotic courses for *Shigella* diarrhea episodes	-6.2 (-7.0, -5.5)	-8.4 (-9.5, -7.4)	0.62 (0.60, 0.64)	0.49 (0.46, 0.52)
Antibiotic courses for diarrhea episodes of any etiology	-6.2 (-7.0, -5.5)	-8.4 (-9.5, -7.4)	0.94 (0.94, 0.95)	0.92 (0.91, 0.93)
Antibiotic courses overall	-6.2 (-7.0, -5.5)	-8.4 (-9.5, -7.4)	0.99 (0.99, 0.99)	0.99 (0.98, 0.99)

VE= vaccine efficacy; CI = confidence interval

Fig B. Relative percent reductions in antibiotic use outcomes (any drug class) among the 9- and 12-month *Shigella* vaccine dosing scenario with 60% full vaccine efficacy against severe *Shigella* diarrhea.

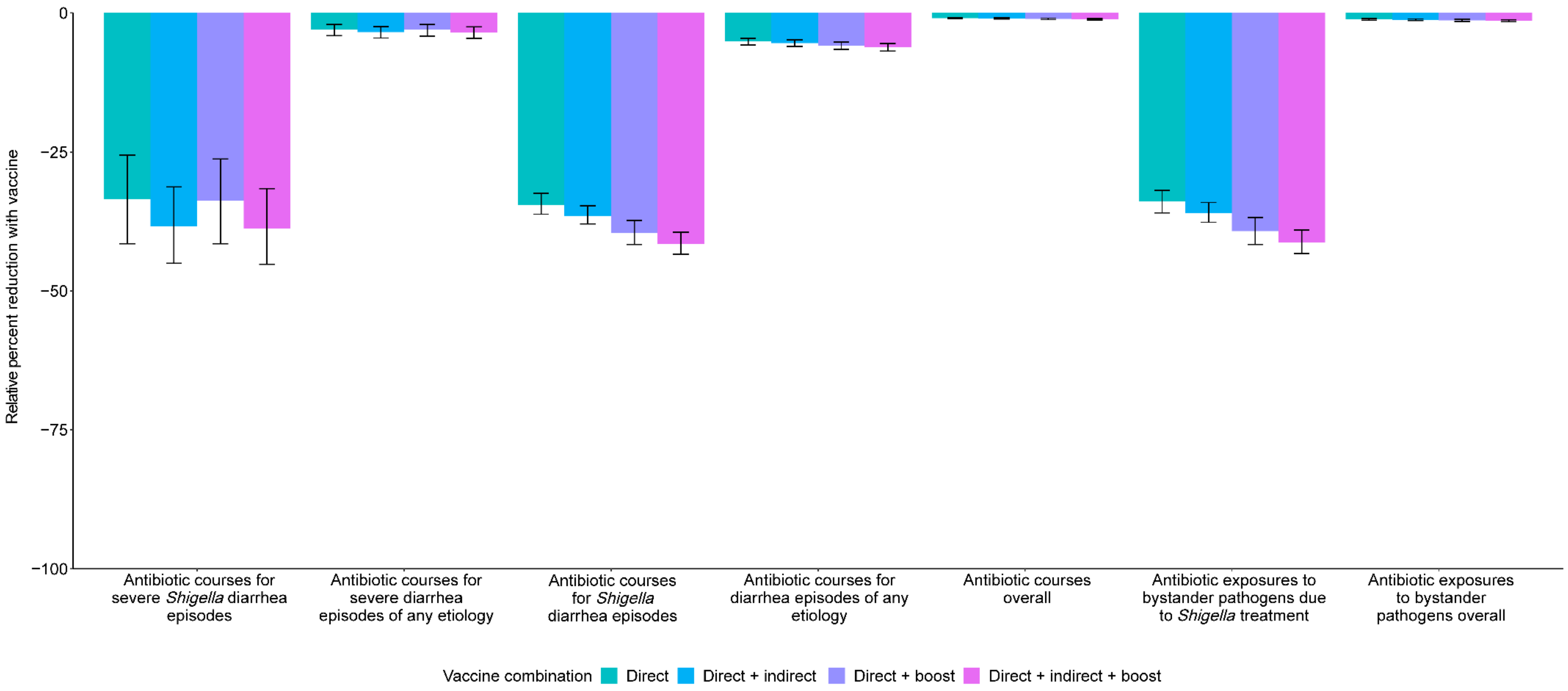

Caption: The black lines represent 95% confidence intervals.

Table P. Relative percent reductions in antibiotic use outcomes (any drug class) among the 9- and 12-month *Shigella* vaccine dosing scenario with 60% full vaccine efficacy against severe *Shigella* diarrhea.

Vaccine scenario and efficacy outcome	Relative percent reduction
Direct	
Antibiotic courses for severe *Shigella* diarrhea episodes	-33.5 (-41.5, -25.6)
Antibiotic courses for severe diarrhea episodes of any etiology	-3.0 (-4.1, -2.1)
Antibiotic courses for *Shigella* diarrhea episodes	-34.5 (-36.2, -32.4)
Antibiotic courses for diarrhea episodes of any etiology	-5.1 (-5.7, -4.6)
Antibiotic courses overall	-0.9 (-1.0, -0.8)
Antibiotic exposures to bystander pathogens due to *Shigella* treatment	-33.9 (-35.9, -31.9)
Antibiotic exposures to bystander pathogens overall	-1.1 (-1.3, -1.0)
Direct + Indirect	
Antibiotic courses for severe *Shigella* diarrhea episodes	-38.4 (-45.0, -31.3)
Antibiotic courses for severe diarrhea episodes of any etiology	-3.4 (-4.5, -2.5)
Antibiotic courses for *Shigella* diarrhea episodes	-36.5 (-37.9, -34.7)
Antibiotic courses for diarrhea episodes of any etiology	-5.4 (-6.0, -4.9)
Antibiotic courses overall	-1.0 (-1.1, -0.9)
Antibiotic exposures to bystander pathogens due to *Shigella* treatment	-36.0 (-37.6, -34.1)
Antibiotic exposures to bystander pathogens overall	-1.2 (-1.4, -1.1)
Direct + Boost	
Antibiotic courses for severe *Shigella* diarrhea episodes	-33.8 (-41.5, -26.2)
Antibiotic courses for severe diarrhea episodes of any etiology	-3.0 (-4.2, -2.1)
Antibiotic courses for *Shigella* diarrhea episodes	-39.6 (-41.6, -37.3)
Antibiotic courses for diarrhea episodes of any etiology	-5.9 (-6.6, -5.2)
Antibiotic courses overall	-1.1 (-1.2, -1.0)
Antibiotic exposures to bystander pathogens due to *Shigella* treatment	-39.2 (-41.6, -36.8)
Antibiotic exposures to bystander pathogens overall	-1.3 (-1.5, -1.2)
Direct + Indirect + Boost	
Antibiotic courses for severe *Shigella* diarrhea episodes	-38.7 (-45.2, -31.6)
Antibiotic courses for severe diarrhea episodes of any etiology	-3.5 (-4.6, -2.5)
Antibiotic courses for *Shigella* diarrhea episodes	-41.5 (-43.4, -39.4)
Antibiotic courses for diarrhea episodes of any etiology	-6.2 (-6.9, -5.5)
Antibiotic courses overall	-1.1 (-1.3, -1.0)
Antibiotic exposures to bystander pathogens due to *Shigella* treatment	-41.3 (-43.3, -39)
Antibiotic exposures to bystander pathogens overall	-1.4 (-1.6, -1.2)

Data are also reported in Fig B. VE= vaccine efficacy; CI = confidence interval

Table Q. Absolute (incidence rate differences) and relative (incidence rate ratios) differences in diarrhea outcomes among the 9- and 12-month *Shigella* vaccine dosing scenario compared to the no vaccine scenario with 60% full vaccine efficacy against severe *Shigella* diarrhea by site.

Vaccine scenario and efficacy outcome	Incidence rate difference (cases per 100 child-years) 60% VE (95% CI)	Incidence rate ratio 60% VE (95% CI)
Bangladesh		
Severe *Shigella* diarrhea episodes	-0.3 (-0.5, -0.1)	0.54 (0.43, 0.66)
Severe diarrhea episodes of any etiology	-0.3 (-0.5, -0.1)	0.96 (0.93, 0.98)
Shigella diarrhea episodes	-3.9 (-4.6, -3.2)	0.65 (0.63, 0.67)
Diarrhea episodes of any etiology	-3.9 (-4.6, -3.2)	0.93 (0.92, 0.94)
Brazil		
Severe *Shigella* diarrhea episodes	--	--
Severe diarrhea episodes of any etiology	--	--
Shigella diarrhea episodes	-0.2 (-0.3, -0.1)	0.60 (0.55, 0.64)
Diarrhea episodes of any etiology	-0.2 (-0.3, -0.1)	0.95 (0.92, 0.97)
India		
Severe *Shigella* diarrhea episodes	-0.2 (-0.4, -0.1)	0.54 (0.42, 0.70)
Severe diarrhea episodes of any etiology	-0.2 (-0.4, -0.1)	0.91 (0.85, 0.96)
Shigella diarrhea episodes	-1.4 (-1.8, -1.1)	0.65 (0.62, 0.69)
Diarrhea episodes of any etiology	-1.4 (-1.8, -1.1)	0.94 (0.93, 0.96)
Nepal		
Severe *Shigella* diarrhea episodes	-0.2 (-0.4, -0.1)	0.40 (0.35, 0.45)
Severe diarrhea episodes of any etiology	-0.2 (-0.4, -0.1)	0.96 (0.92, 0.98)
Shigella diarrhea episodes	-1.3 (-1.6, -1.0)	0.60 (0.57, 0.64)
Diarrhea episodes of any etiology	-1.3 (-1.6, -1.0)	0.97 (0.96, 0.97)
Pakistan		
Severe *Shigella* diarrhea episodes	-0.5 (-0.7, -0.3)	0.62 (0.53, 0.71)
Severe diarrhea episodes of any etiology	-0.5 (-0.7, -0.3)	0.98 (0.97, 0.99)
Shigella diarrhea episodes	-1.4 (-1.8, -1.0)	0.66 (0.63, 0.70)
Diarrhea episodes of any etiology	-1.4 (-1.8, -1.0)	0.98 (0.98, 0.99)
Peru		
Severe *Shigella* diarrhea episodes	-0.3 (-0.5, -0.2)	0.40 (0.36, 0.44)
Severe diarrhea episodes of any etiology	-0.3 (-0.5, -0.2)	0.95 (0.92, 0.97)
Shigella diarrhea episodes	-2.6 (-3.2, -2.0)	0.61 (0.59, 0.63)
Diarrhea episodes of any etiology	-2.6 (-3.2, -2.0)	0.96 (0.95, 0.97)
South Africa		
Severe *Shigella* diarrhea episodes	0.0 (0.0, 0.0)	0.71 (0.57, 0.83)
Severe diarrhea episodes of any etiology	0.0 (0.0, 0.0)	0.93 (0.70, 1.00)
Shigella diarrhea episodes	-0.1 (-0.2, -0.1)	0.69 (0.60, 0.79)
Diarrhea episodes of any etiology	-0.1 (-0.2, -0.1)	0.97 (0.95, 0.99)
Tanzania		
Severe *Shigella* diarrhea episodes	--	--
Severe diarrhea episodes of any etiology	--	--
Shigella diarrhea episodes	-0.1 (-0.1, 0.0)	0.87 (0.76, 0.98)
Diarrhea episodes of any etiology	-0.1 (-0.1, 0.0)	0.99 (0.98, 1.00)

VE= vaccine efficacy; CI = confidence interval

Fig C. Relative percent reductions in diarrhea outcomes among the 9- and 12-month *Shigella* vaccine dosing scenario with 60% full vaccine efficacy against severe *Shigella* diarrhea by site.

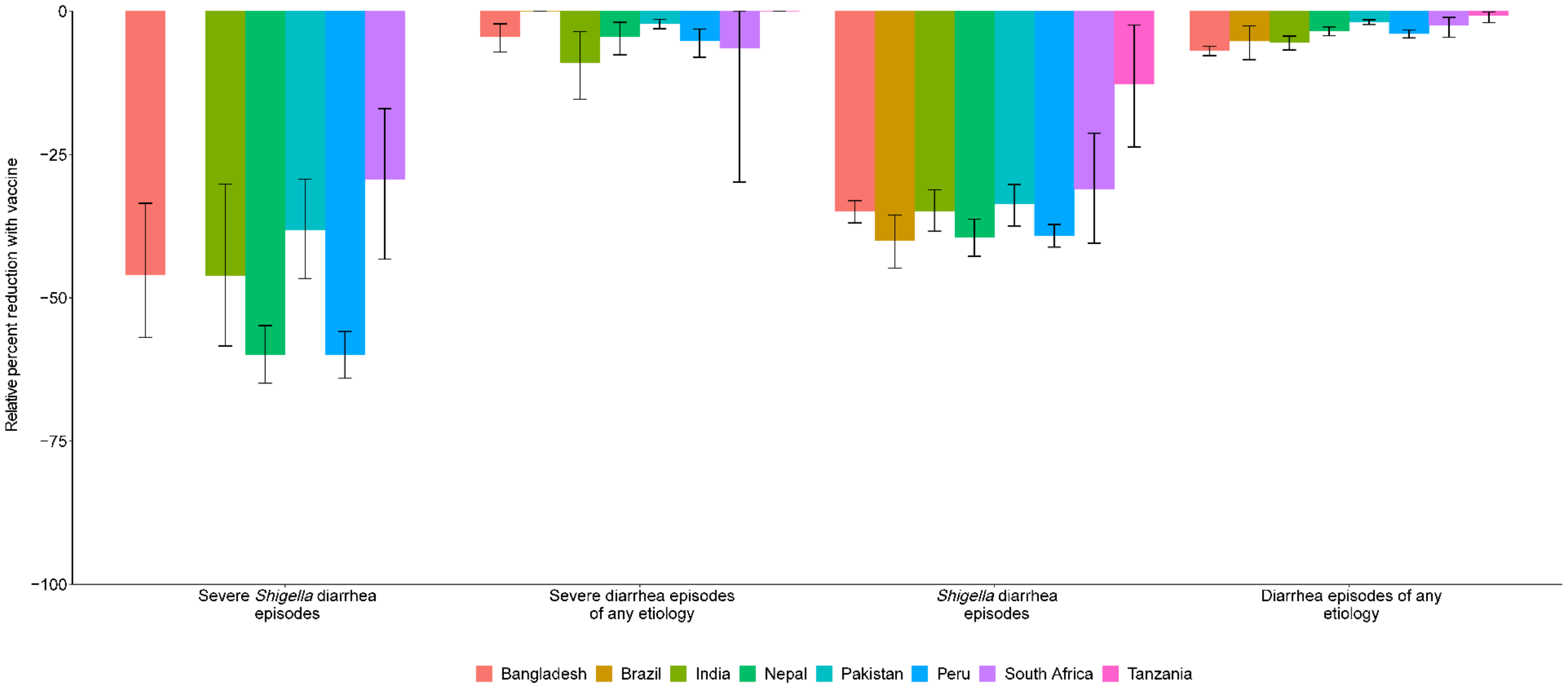

Caption: The black lines represent 95% confidence intervals.

Table R. Absolute (incidence rate differences) and relative (incidence rate ratios) differences in fluoroquinolone/macrolide (F/M) outcomes among the 9- and 12-month *Shigella* vaccine dosing scenario compared to the no vaccine scenario with 60% full vaccine efficacy against severe *Shigella* diarrhea by site.

Vaccine scenario and efficacy outcome	Incidence rate difference (cases per 100 child-years) 60% VE (95% CI)	Incidence rate ratio 60% VE (95% CI)
Bangladesh		
F/M courses for severe *Shigella* diarrhea episodes	-0.2 (-0.4, -0.1)	0.58 (0.44, 0.80)
F/M courses for severe diarrhea episodes of any etiology	-0.2 (-0.4, -0.1)	0.94 (0.89, 0.98)
F/M courses for *Shigella* diarrhea episodes	-2.0 (-2.4, -1.6)	0.64 (0.61, 0.67)
F/M courses for diarrhea episodes of any etiology	-2.0 (-2.4, -1.6)	0.91 (0.89, 0.92)
F/M courses overall	-2.0 (-2.4, -1.6)	0.96 (0.96, 0.97)
F/M exposures to bystander pathogens due to *Shigella* treatment	-4.1 (-5.1, -3.2)	0.65 (0.62, 0.68)
F/M exposures to bystander pathogens overall	-4.1 (-5.1, -3.2)	0.96 (0.95, 0.97)
Brazil		
F/M courses for severe *Shigella* diarrhea episodes	--	--
F/M courses for severe diarrhea episodes of any etiology	--	--
F/M courses for *Shigella* diarrhea episodes	--	--
F/M courses for diarrhea episodes of any etiology	--	--
F/M courses overall	--	--
F/M exposures to bystander pathogens due to *Shigella* treatment	--	--
F/M exposures to bystander pathogens overall	--	--
India		
F/M courses for severe *Shigella* diarrhea episodes	-0.1 (-0.2, 0.0)	0.54 (0.31, 1.00)
F/M courses for severe diarrhea episodes of any etiology	-0.1 (-0.2, 0.0)	0.83 (0.64, 1.00)
F/M courses for *Shigella* diarrhea episodes	-0.1 (-0.2, 0.0)	0.64 (0.47, 0.82)
F/M courses for diarrhea episodes of any etiology	-0.1 (-0.2, 0.0)	0.94 (0.89, 0.98)
F/M courses overall	-0.1 (-0.2, 0.0)	0.99 (0.98, 1.00)
F/M exposures to bystander pathogens due to *Shigella* treatment	-0.2 (-0.4, -0.1)	0.70 (0.49, 0.86)
F/M exposures to bystander pathogens overall	-0.2 (-0.4, -0.1)	0.99 (0.97, 1.00)
Nepal		
F/M courses for severe *Shigella* diarrhea episodes	0.0 (-0.1, 0.0)	0.40 (0.27, 0.54)
F/M courses for severe diarrhea episodes of any etiology	0.0 (-0.1, 0.0)	0.89 (0.69, 1.00)
F/M courses for *Shigella* diarrhea episodes	-0.1 (-0.3, -0.1)	0.56 (0.48, 0.64)
F/M courses for diarrhea episodes of any etiology	-0.1 (-0.3, -0.1)	0.90 (0.83, 0.95)

F/M courses overall	-0.1 (-0.3, -0.1)	0.98 (0.97, 0.99)
F/M exposures to bystander pathogens due to *Shigella* treatment	-0.2 (-0.4, -0.1)	0.58 (0.49, 0.66)
F/M exposures to bystander pathogens overall	-0.2 (-0.4, -0.1)	0.99 (0.97, 0.99)
Pakistan		
F/M courses for severe *Shigella* diarrhea episodes	--	--
F/M courses for severe diarrhea episodes of any etiology	--	--
F/M courses for *Shigella* diarrhea episodes	0.0 (-0.1, 0.0)	0.74 (0.51, 1.00)
F/M courses for diarrhea episodes of any etiology	0.0 (-0.1, 0.0)	0.99 (0.96, 1.00)
F/M courses overall	0.0 (-0.1, 0.0)	1.00 (0.99, 1.00)
F/M exposures to bystander pathogens due to *Shigella* treatment	-0.1 (-0.3, 0.0)	0.69 (0.51, 1.00)
F/M exposures to bystander pathogens overall	-0.1 (-0.3, 0.0)	1.00 (0.99, 1.00)
Peru		
F/M courses for severe *Shigella* diarrhea episodes	-0.1 (-0.3, -0.1)	0.40 (0.34, 0.47)
F/M courses for severe diarrhea episodes of any etiology	-0.1 (-0.3, -0.1)	0.92 (0.86, 0.97)
F/M courses for *Shigella* diarrhea episodes	-0.7 (-0.9, -0.5)	0.59 (0.56, 0.63)
F/M courses for diarrhea episodes of any etiology	-0.7 (-0.9, -0.5)	0.93 (0.91, 0.95)
F/M courses overall	-0.7 (-0.9, -0.5)	0.96 (0.95, 0.97)
F/M exposures to bystander pathogens due to *Shigella* treatment	-1.4 (-1.9, -0.9)	0.59 (0.55, 0.64)
F/M exposures to bystander pathogens overall	-1.4 (-1.9, -0.9)	0.96 (0.95, 0.97)
South Africa		
F/M courses for severe *Shigella* diarrhea episodes	--	--
F/M courses for severe diarrhea episodes of any etiology	--	--
F/M courses for *Shigella* diarrhea episodes	--	--
F/M courses for diarrhea episodes of any etiology	--	--
F/M courses overall	--	--
F/M exposures to bystander pathogens due to *Shigella* treatment	--	--
F/M exposures to bystander pathogens overall	--	--
Tanzania		
F/M courses for severe *Shigella* diarrhea episodes	--	--
F/M courses for severe diarrhea episodes of any etiology	--	--
F/M courses for *Shigella* diarrhea episodes	--	--
F/M courses for diarrhea episodes of any etiology	--	--
F/M courses overall	--	--
F/M exposures to bystander pathogens due to *Shigella* treatment	--	--
F/M exposures to bystander pathogens overall	--	--

VE= vaccine efficacy; CI = confidence interval; F/M = fluoroquinolones/macrolides

Fig D. Relative percent reductions in fluoroquinolone and macrolide (F/M) use among the 9- and 12-month *Shigella* vaccine dosing scenario with 60% full vaccine efficacy against severe *Shigella* diarrhea by site.

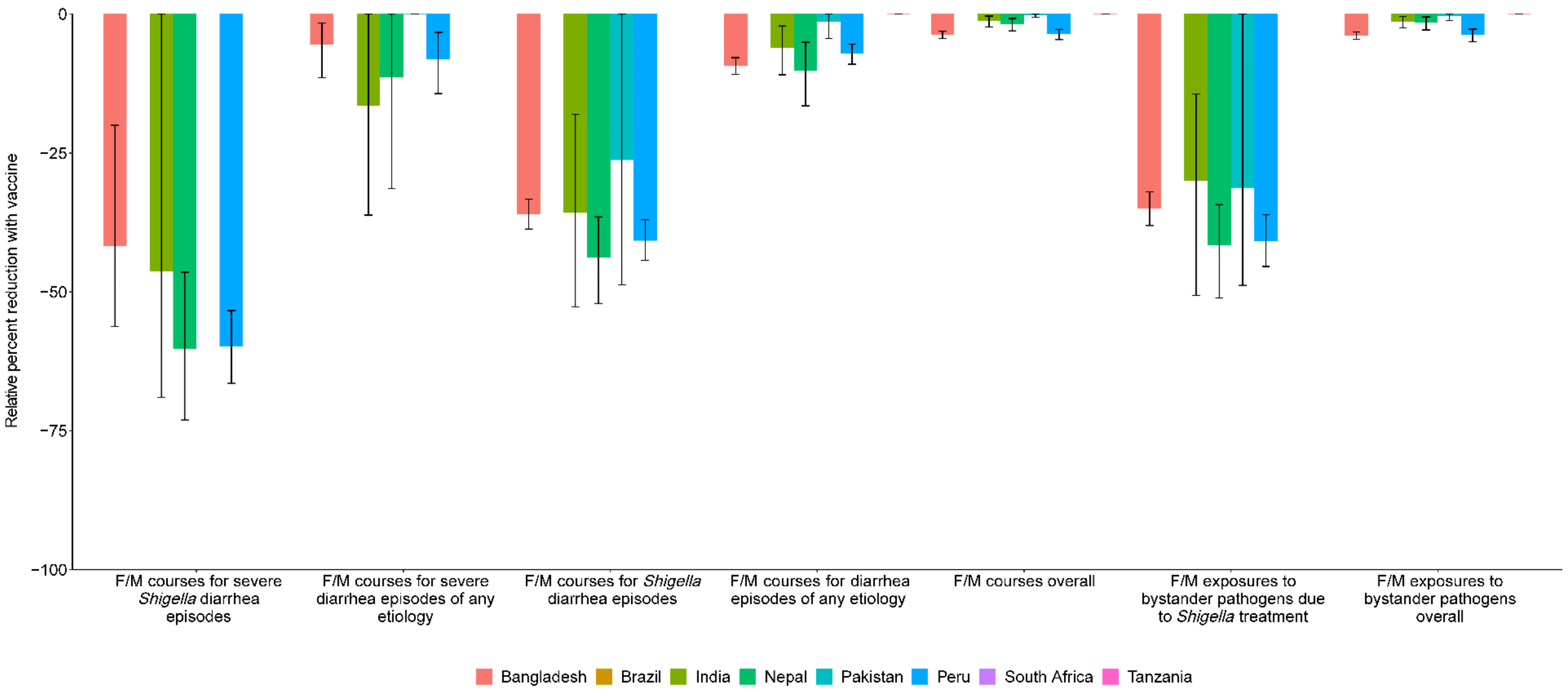

Caption: The black lines represent 95% confidence intervals.

Table S. Absolute (incidence rate differences) and relative (incidence rate ratios) differences in antibiotic use outcomes (any drug class) among the 9- and 12-month *Shigella* vaccine dosing scenario compared to the no vaccine scenario with 60% full vaccine efficacy against severe *Shigella* diarrhea by site.

Vaccine scenario and efficacy outcome	Incidence rate difference (cases per 100 child-years) 60% VE (95% CI)	Incidence rate ratio 60% VE (95% CI)
Bangladesh		
Antibiotic courses for severe *Shigella* diarrhea episodes	-0.2 (-0.5, -0.1)	0.58 (0.43, 0.78)
Antibiotic courses for severe diarrhea episodes of any etiology	-0.2 (-0.5, -0.1)	0.94 (0.90, 0.98)
Antibiotic courses for *Shigella* diarrhea episodes	-2.2 (-2.7, -1.8)	0.64 (0.62, 0.67)
Antibiotic courses for diarrhea episodes of any etiology	-2.2 (-2.7, -1.8)	0.91 (0.90, 0.93)
Antibiotic courses overall	-2.2 (-2.7, -1.8)	0.98 (0.98, 0.98)
Antibiotic exposures to bystander pathogens due to *Shigella* treatment	-4.7 (-5.8, -3.6)	0.65 (0.63, 0.68)
Antibiotic exposures to bystander pathogens overall	-4.7 (-5.8, -3.6)	0.98 (0.97, 0.98)
Brazil		
Antibiotic courses for severe *Shigella* diarrhea episodes	--	--
Antibiotic courses for severe diarrhea episodes of any etiology	--	--
Antibiotic courses for *Shigella* diarrhea episodes	0.0 (-0.1, 0.0)	0.60 (0.43, 0.74)
Antibiotic courses for diarrhea episodes of any etiology	0.0 (-0.1, 0.0)	0.97 (0.87, 1.00)
Antibiotic courses overall	0.0 (-0.1, 0.0)	1.00 (0.99, 1.00)
Antibiotic exposures to bystander pathogens due to *Shigella* treatment	--	--
Antibiotic exposures to bystander pathogens overall	--	--
India		
Antibiotic courses for severe *Shigella* diarrhea episodes	-0.1 (-0.2, 0.0)	0.66 (0.41, 0.86)
Antibiotic courses for severe diarrhea episodes of any etiology	-0.1 (-0.2, 0.0)	0.93 (0.85, 0.98)
Antibiotic courses for *Shigella* diarrhea episodes	-0.4 (-0.6, -0.2)	0.66 (0.59, 0.74)
Antibiotic courses for diarrhea episodes of any etiology	-0.4 (-0.6, -0.2)	0.93 (0.90, 0.96)
Antibiotic courses overall	-0.4 (-0.6, -0.2)	0.99 (0.99, 1.00)
Antibiotic exposures to bystander pathogens due to *Shigella* treatment	-0.6 (-0.9, -0.3)	0.69 (0.60, 0.79)
Antibiotic exposures to bystander pathogens overall	-0.6 (-0.9, -0.3)	1.00 (0.99, 1.00)
Nepal		
Antibiotic courses for severe *Shigella* diarrhea episodes	-0.1 (-0.3, 0.0)	0.40 (0.33, 0.48)
Antibiotic courses for severe diarrhea episodes of any etiology	-0.1 (-0.3, 0.0)	0.92 (0.85, 0.98)
Antibiotic courses for *Shigella* diarrhea episodes	-0.6 (-0.8, -0.4)	0.61 (0.56, 0.66)
Antibiotic courses for diarrhea episodes of any etiology	-0.6 (-0.8, -0.4)	0.93 (0.90, 0.95)

Antibiotic courses overall	-0.6 (-0.8, -0.4)	0.98 (0.98, 0.99)
Antibiotic exposures to bystander pathogens due to *Shigella* treatment	-1.0 (-1.4, -0.6)	0.61 (0.56, 0.67)
Antibiotic exposures to bystander pathogens overall	-1.0 (-1.4, -0.6)	0.98 (0.97, 0.99)
Pakistan		
Antibiotic courses for severe *Shigella* diarrhea episodes	-0.3 (-0.5, -0.2)	0.70 (0.57, 0.81)
Antibiotic courses for severe diarrhea episodes of any etiology	-0.3 (-0.5, -0.2)	0.98 (0.97, 0.99)
Antibiotic courses for *Shigella* diarrhea episodes	-0.8 (-1.1, -0.5)	0.69 (0.63, 0.74)
Antibiotic courses for diarrhea episodes of any etiology	-0.8 (-1.1, -0.5)	0.98 (0.97, 0.99)
Antibiotic courses overall	-0.8 (-1.1, -0.5)	1.00 (1.00, 1.00)
Antibiotic exposures to bystander pathogens due to *Shigella* treatment	-0.9 (-1.4, -0.6)	0.70 (0.62, 0.77)
Antibiotic exposures to bystander pathogens overall	-0.9 (-1.4, -0.6)	1.00 (0.99, 1.00)
Peru		
Antibiotic courses for severe *Shigella* diarrhea episodes	-0.2 (-0.3, -0.1)	0.40 (0.34, 0.45)
Antibiotic courses for severe diarrhea episodes of any etiology	-0.2 (-0.3, -0.1)	0.94 (0.90, 0.97)
Antibiotic courses for *Shigella* diarrhea episodes	-1.1 (-1.4, -0.8)	0.60 (0.57, 0.63)
Antibiotic courses for diarrhea episodes of any etiology	-1.1 (-1.4, -0.8)	0.94 (0.93, 0.96)
Antibiotic courses overall	-1.1 (-1.4, -0.8)	0.98 (0.98, 0.99)
Antibiotic exposures to bystander pathogens due to *Shigella* treatment	-2.2 (-2.9, -1.6)	0.59 (0.56, 0.63)
Antibiotic exposures to bystander pathogens overall	-2.2 (-2.9, -1.6)	0.98 (0.98, 0.99)
South Africa		
Antibiotic courses for severe *Shigella* diarrhea episodes	--	--
Antibiotic courses for severe diarrhea episodes of any etiology	--	--
Antibiotic courses for *Shigella* diarrhea episodes	0.0 (-0.1, 0.0)	0.81 (0.51, 1.00)
Antibiotic courses for diarrhea episodes of any etiology	0.0 (-0.1, 0.0)	0.99 (0.95, 1.00)
Antibiotic courses overall	0.0 (-0.1, 0.0)	1.00 (1.00, 1.00)
Antibiotic exposures to bystander pathogens due to *Shigella* treatment	--	--
Antibiotic exposures to bystander pathogens overall	--	--
Tanzania		
Antibiotic courses for severe *Shigella* diarrhea episodes	--	--
Antibiotic courses for severe diarrhea episodes of any etiology	--	--
Antibiotic courses for *Shigella* diarrhea episodes	-0.1 (-0.2, 0.0)	0.88 (0.73, 1.00)
Antibiotic courses for diarrhea episodes of any etiology	-0.1 (-0.2, 0.0)	0.99 (0.97, 1.00)
Antibiotic courses overall	-0.1 (-0.2, 0.0)	1.00 (1.00, 1.00)
Antibiotic exposures to bystander pathogens due to *Shigella* treatment	-0.2 (-0.5, 0.0)	0.91 (0.74, 1.00)
Antibiotic exposures to bystander pathogens overall	-0.2 (-0.5, 0.0)	1.00 (1.00, 1.00)

VE= vaccine efficacy; CI = confidence interval
